# Novel interaction interfaces mediate the interaction between the NEIL1 DNA glycosylase and mitochondrial transcription factor A

**DOI:** 10.3389/fcell.2022.893806

**Published:** 2022-07-22

**Authors:** Nidhi Sharma, Marlo K. Thompson, Jennifer F. Arrington, Dava M. Terry, Srinivas Chakravarthy, Peter E. Prevelige, Aishwarya Prakash

**Affiliations:** ^1^ Department of Biochemistry and Molecular Biology, Mitchell Cancer Institute, University of South Alabama, Mobile, AL, United States; ^2^ Advanced Photon Source, Illinois Institute of Technology, Chicago, IL, United States; ^3^ Department of Microbiology, University of Alabama at Birmingham, Birmingham, AL, United States

**Keywords:** base excision repair (BER), NEIL1 DNA glycosylase, mitochondrial transcription factor A (TFAM), small angle X-ray scattering (SAXS), HDX-MS (hydrogen–deuterium exchange mass-spectrometry)

## Abstract

The maintenance of human mitochondrial DNA (mtDNA) is critical for proper cellular function as damage to mtDNA, if left unrepaired, can lead to a diverse array of pathologies. Of the pathways identified to participate in DNA repair within the mitochondria, base excision repair (BER) is the most extensively studied. Protein-protein interactions drive the step-by-step coordination required for the successful completion of this pathway and are important for crosstalk with other mitochondrial factors involved in genome maintenance. Human NEIL1 is one of seven DNA glycosylases that initiates BER in both the nuclear and mitochondrial compartments. In the current work, we scrutinized the interaction between NEIL1 and mitochondrial transcription factor A (TFAM), a protein that is essential for various aspects of mtDNA metabolism. We note, for the first time, that both the N- and C- terminal domains of NEIL1 interact with TFAM revealing a unique NEIL1 protein-binding interface. The interaction between the two proteins, as observed biochemically, appears to be transient and is most apparent at concentrations of low salt. The presence of DNA (or RNA) also positively influences the interaction between the two proteins, and molar mass estimates indicate that duplex DNA is required for complex formation at higher salt concentrations. Hydrogen deuterium exchange mass spectrometry data reveal that both proteins exchange less deuterium upon DNA binding, indicative of an interaction, and the addition of NEIL1 to the TFAM-DNA complex alters the interaction landscape. The transcriptional activity of TFAM appears to be independent of NEIL1 expression under normal cellular conditions, however, in the presence of DNA damage, we observe a significant reduction in the mRNA expression of TFAM-transcribed mitochondrial genes in the absence of NEIL1. Overall, our data indicate that the interaction between NEIL1 and TFAM can be modulated by local environment such as salt concentrations, protein availability, the presence of nucleic acids, as well as the presence of DNA damage.

## Introduction

Maintaining the integrity of the 16.5 kb circular human mitochondrial genome is essential for proper cellular function as mitochondrial DNA (mtDNA) encodes for 13 polypeptides, 22 tRNAs, and two rRNAs that are required for the generation of ATP *via* oxidative phosphorylation (OXPHOS) (Anderson et al., 1981). Mitochondria rely heavily upon nuclear-encoded proteins for genome maintenance. The mitochondrial proteome comprises some ∼1,500 proteins that are transported to the organelle *via* different import mechanisms (Endo et al., 2011; Wiedemann and Pfanner, 2017; Ruan et al., 2020). Much like its nuclear counterpart, mtDNA is also subjected to damage from various endogenous and exogenous sources that leads to genome instability and various pathologies, including neurodegenerative disorders, metabolic dysfunction, and some cancers (Copeland and Longley, 2014; Van Houten et al., 2016; Rahman and Copeland, 2019). MtDNA molecules were originally thought to be especially prone to damage by reactive oxygen species (ROS) owing to their lack of protection from nucleosomes as well as their location proximal to sites of OXPHOS within the mitochondrial inner membrane; however, this assumption has recently been contested as mtDNA are compacted into nucleoid complexes (further discussed below) and ROS appear to be concentrated within the mitochondrial cristae (Yakes and Van Houten, 1997; Anson et al., 2000; Lim et al., 2005; Kauppila and Stewart, 2015). However, to cope with mtDNA damage, several nuclear-encoded repair factors that participate in multiple DNA repair pathways have been identified in the mitochondria. Of these, the mitochondrial base excision repair (mtBER) pathway appears to be the major pathway involved with the removal of small, non-bulky lesions that arise in the DNA (Fromme and Verdine, 2004; Szczesny et al., 2008; Wallace et al., 2012; Krokan and Bjoras, 2013; Prakash and Doublié, 2015). Several nuclear BER enzymes have been implicated in the repair of mtDNA, including seven of the eleven known mammalian DNA glycosylases that initiate BER, end processing enzymes such as polynucleotide kinase phosphate (PNKP), DNA polymerases such as polymerase beta (Polβ) that are needed to fill in the gap generated, and DNA ligase III needed to seal the gap (Prakash and Doublié, 2015; Saki and Prakash, 2017; Baptiste et al., 2021).

The NEIL1 enzyme is a bifunctional DNA glycosylase that possesses both glycosylase and lyase functions and is involved with the removal of oxidized DNA bases such as thymine glycol, 5-hydroxy uracil, the ring-opened formamidopyrimidine (Fapy) lesions, as well as the further oxidation products of 8-oxo guanine (8-oxoG), namely spiroiminodihydantoin (Sp) and guanidinohydantoin (Gh) that result in an abasic (AP) site (Krishnamurthy et al., 2008; Zhao et al., 2010; Prakash et al., 2012; Vik et al., 2012; Minko et al., 2020; Yeo et al., 2021). NEIL1 also possesses lyase activity by which it can incise the DNA backbone at AP sites. Crystal structures of NEIL1 unliganded and bound to DNA containing oxidized DNA lesions have provided valuable insight into the enzymatic and lesion recognition activity of the enzyme (Doublié et al., 2004; Zhu et al., 2016; Liu et al., 2021). Notably, the structures of NEIL1 lacked a large region of disorder at the C-terminal end of the protein. Previously, we and others have shown that this disordered tail of NEIL1 comprising 100 amino acid residues (aa 290–390) participates in the interaction with its binding partners, including the homotrimeric proliferating cellular nuclear antigen (PCNA), flap endonuclease 1 (FEN-1), heterotrimeric replication protein A (RPA), the Werner Syndrome RecQ like helicase (WRN helicase), and mitochondrial single stranded DNA binding protein (mtSSB) (Das et al., 2007; Dou et al., 2008; Hegde et al., 2008; Theriot et al., 2010; Prakash et al., 2017; Sharma et al., 2018).

Unlike nuclear DNA that is packaged into nucleosomes, mtDNA is packaged into nucleo-protein structures called nucleoids, which are approximately 110 nm in size based on high-resolution techniques (Kukat et al., 2011; Bogenhagen, 2012; Campbell et al., 2012; Farge and Falkenberg, 2019). The mitochondrial transcription factor A (TFAM) protein, a major component of mitochondrial nucleoids was originally thought to be sufficient for the higher-order organization of the mitochondrial genome and is also required to initiate transcription from the light and heavy strand promoter regions of mtDNA (Alam et al., 2003; Kaufman et al., 2007; Kukat et al., 2015). However, recent reports [reviewed in (Mishmar et al., 2019)] implicate nuclear factors such as MOF in the binding and regulation of higher-order genome organization within the mitochondrion, suggesting a more complex scenario involving nuclear-mitochondrial crosstalk. Although current experimental strategies limit the examination of the exact composition of the mitochondrial nucleoid, several other proteins, including mtSSB, the mtDNA replicative polymerase gamma (POLG), and the twinkle helicase, are associated with the mitochondrial nucleoid (Bogenhagen et al., 2008).

Evidence for the participation of TFAM in mtBER was provided by studies indicating the ability of TFAM to bind to 8-oxoG while inhibiting BER enzymes, including 8-oxoG DNA glycosylase (OGG1), uracil-DNA glycosylase (UDG), and apurinic endonuclease 1 (APE1) (Canugovi et al., 2010). TFAM was further implicated in the cleavage of AP sites to yield single-strand breaks in the DNA (Xu et al., 2019). Given the substrate overlap between NEIL1 and TFAM and the likely close proximity of NEIL1 to the mitochondrial nucleoid, in the current manuscript, we report for the first time an interaction between NEIL1 and TFAM *via* unique interaction interfaces. While the interaction between the two proteins appears to be transient and dependent upon the presence of DNA, we noted that NEIL1 can interact with TFAM using multiple regions within both its N- and C- termini. This finding is novel and represents a deviation from previous observations that suggest the interaction domain of NEIL1 resides solely within its C-terminal disordered tail. Here, we use orthogonal approaches including far-western analysis, pull-down studies, small-angle X-ray scattering (SAXS), multi-angle light scattering (MALS), and hydrogen deuterium exchange mass spectrometry (HDX-MS), to probe the interaction between the two proteins. Our data collectively suggest that the interaction between NEIL1 and TFAM is transient and can be enhanced by modulating the local environment such as salt concentration, protein availability, and the presence of DNA or RNA. Furthermore, the importance of the interaction between the two proteins is underscored by the observation that NEIL1 is required for the transcription of mitochondrial genes under conditions of cellular stress.

## Experimental approach

### Plasmids, DNA, and RNA oligonucleotides

The bacterial expression plasmid containing C-terminal His-tagged NEIL1 (pET30a) was obtained from the laboratories of Dr. Sylvie Doublié and Dr. Susan Wallace (University of Vermont, United States). The plasmid containing the ORF for full-length TFAM was a kind gift from Dr. Robert Sobol (University of South Alabama, United States). The TFAM DNA sequence lacking the mitochondrial targeting sequence was amplified from this plasmid and subcloned into a pET30a vector. The *E. coli* expression plasmids containing the full-length (FL) NEIL1-FL, C-terminal truncated polypeptides (∆40, ∆56, ∆78, and ∆100), and GST fused C-terminal regions (289–389, 289–349, 312–389, and 312–349) were synthesized as described previously (Das et al., 2007; Prakash et al., 2017; Sharma et al., 2018). The Flag peptide sequence was included at the C-terminus of both NEIL1-FL and TFAM in the pET30a background using primers containing the Flag nucleotide sequence by traditional cloning methods with restriction enzyme digestion.

The duplex oligonucleotide substrate used in these studies is a 22-nt oligomer 5′-ATTCAACCAA**X**AGCCCTGGCCG-3′ with a complementary oligomer 5′-CGG​CCA​GGG​CTA​TTG​GTT​GAA​T-3′. The X represents either tetrahydrofuran, an abasic site analog (specific DNA; SD), or thymine (nonspecific DNA; NSD). The oligos were ordered from Integrated DNA Technologies Inc. (IDT, Coralville, IA) and PAGE purified. For optimal annealing, equimolar mixtures of the oligomers were heated at 94°C for 2 min, then slowly cooled to room temperature. A 22-nt single-stranded RNA oligonucleotide with the sequence 5′-rArUrUrCrArArCrCrArA**Xr**ArGrCrCrCrUrGrGrCrCrG-3′ was synthesized by IDT for use in the affinity pull-down experiments.

### Overexpression and purification of NEIL1 and TFAM

TFAM was overexpressed and purified as described previously (Ngo et al., 2011). NEIL1-FL, the C-terminal truncated polypeptides, and the GST fused NEIL1 C-terminal regions were overexpressed and purified as described previously (Sharma et al., 2018). Briefly, protein expression was induced by 0.4–1 mM IPTG at 18–25°C in the Rosetta 2 *E. coli* expression strain. Bacterial pellets were lysed using sonication, and the resulting cell debris was discarded after centrifugation at 25,000 × g for 1 h. Protein purification was performed using affinity chromatography (Talon beads or Glutathione S Sepharose) following the manufacturer’s protocol (Clontech Laboratories, Inc., Mountain View, CA; and Millipore-Sigma, St. Louis, MO). The proteins were further purified using a HiTrap SPFF ion exchange column and Superdex 200 Increase 10/300 GL size exclusion column (Cytiva). The purified fractions were concentrated and stored at -80°C until further use.

### Far-western analysis

Far-western analysis was performed as described previously (Prakash et al., 2017). Briefly, proteins (50 pmol of the NEIL1 polypeptides, bovine serum albumin, and glutathione S-transferase; 1 pmol TFAM) were separated by 12% SDS-PAGE, transferred to a PVDF membrane (BioRad), treated with 6M guanidine HCl in PBS containing 1 mM DTT, and then gradually refolded with successive dilutions of guanidine HCl in PBS containing 1 mM DTT. The membranes were then blocked for 3 h with 5% milk in PBS at 4°C and incubated with purified TFAM (1 pmol/ml) overnight. Immunoblot analysis was performed using an anti-TFAM antibody (Cell signaling #7495) at 1:1,000 dilution in 5% BSA in PBST.

### Affinity pull-down using Flag-tagged recombinant protein

Affinity pull-down experiments were performed using C-terminal Flag-tagged purified NEIL1 or TFAM in wash buffer containing 50 mM Tris-HCl pH 7.5, 10% glycerol, and 50–300 mM NaCl/KCl. Briefly, 1.25/2.5 nM each of NEIL1, TFAM, and specific DNA or RNA were mixed in a final volume of 400 µl in wash buffer and incubated on ice for 1 h. The mixture was then added to 20 µl Anti-FLAG M2 Magnetic Beads (#M8823 Millipore Sigma), pre-equilibrated with wash buffer, and tumbled end over end for 2 h at 4°C. The beads were then washed with 250 µl wash buffer three times, followed by elution using 3X Flag peptide (#F4799 Millipore Sigma). The samples were analyzed *via* SDS-PAGE. For analysis of complex formation in the presence of Benzonase Nuclease (Millipore-Sigma catalog #E1014) where indicated, 125 units of Benzonase Nuclease (at 250 units/µl) was added to the proteins prior to complex formation.

### Complex formation and size exclusion chromatography

The NEIL1-TFAM complex was prepared by mixing both proteins in a 1:1 M ratio in a buffer containing 20 mM HEPES (pH 7.5), 300 mM NaCl, 10% (v/v) glycerol, and 1 mM DTT. The complex was incubated on ice for 1 h prior to size exclusion chromatography (SEC) analysis. The ternary complex of TFAM-NEIL1-DNA was prepared by mixing each in a 1:1:1 M ratio followed by incubation on ice for 1 h. The SEC was performed with a Superdex 200 column using a buffer containing 20 mM HEPES (pH 7.5), 300 mM NaCl, 10% (v/v) glycerol, and 1 mM DTT. The NEIL1-DNA and TFAM-DNA complexes were prepared by mixing the proteins and the DNA in a 1:1 M ratio and incubated for 1 h on ice prior to loading onto the SEC column. The column was calibrated with blue dextran (to determine the void volume) and three standards of known molecular weights (MW) using Gel Filtration LMW Calibration Kit (Cytiva # 28403841).

### Small-angle X-ray scattering and multi-angle light scattering

SEC-MALS-SAXS/SEC-SAXS data were collected at beamline 18-ID (BioCAT) of the Advanced Photon Source (APS) at Argonne National Laboratory. All experiments were performed in the buffer containing 25 mM HEPES pH 7.4, 300 mM NaCl, 5% glycerol, and 1 mM DTT. Samples for data collection included individual proteins (NEIL1-FL and TFAM), the NEIL1-TFAM complex, protein-DNA complexes (NEIL1-DNA and TFAM-DNA), and the ternary complex (TFAM-NEIL1-DNA) at concentrations of 7–11 mg/ml (190 µM each) at 300 µl volume. The samples were loaded onto an in-line SEC column (Superdex 200 10/300) coupled to a MALS detector (DAWN Helios II, Wyatt Technologies) and a SAXS flow cell. At a flow rate of 0.7 ml/min, 0.5 s exposures were acquired every 1 s. The SEC-SAXS data files were processed in BioXTAS RAW (version 2.1.0) using evolving factor analysis (EFA) to extract scattering profiles for each component in overlapping peaks (Meisburger et al., 2016; Hopkins et al., 2017). Forward scattering intensity (I_0_) and radius of gyration (R_g_) were determined using Guinier fit (Konarev et al., 2003). The Kratky plots were normalized against Rg using BioXTAS RAW. Scattering curves were further analyzed using GNOM for the calculation of I_0_, R_g_, distance distribution P(r), maximum dimension (D_max_), Porod volume (V_ρ_), and excluded volume (V_e_) (Svergun, 1992). The MW values were estimated using volume of correlation, Porod volume, ATSAS datclass/ShapeandSize, and Bayesian estimation methods (Rambo and Tainer, 2013; Franke et al., 2018; Hajizadeh et al., 2018; Piiadov et al., 2019). Absolute molar mass values were also calculated from MALS for comparison with the SAXS MW estimates using the ASTRA software (Wyatt Technologies).

### Hydrogen deuterium exchange-mass spectrometry

HDX-MS experiments were performed on a Synapt G2-Si (Waters Corp.) and a Leap HD/X-PAL (Trajan) fluidics system. The proteins and DNA were mixed in an equimolar ratio at a concentration of 100 µM and incubated for 1 h in a buffer containing 50 mM Tris-HCl pH 7.5, 10% glycerol, 100 mM NaCl, and 1 mM DTT. Deuterium exchange was initiated by diluting the samples 10X to a final concentration of 10 µM in an equivalent buffer made with D_2_O at 20°C. After incubation of protein samples for different time points (15, 30, 60, 90 s, 3, 10, and 30 m), the exchange reaction was stopped, and in-solution digestion (2 min at 2°C) was initiated by 10X dilution into 0.3 mg/ml pepsin in 100 mM potassium phosphate buffer pH 2.5. Non-deuterated protein samples for control measurements were also prepared following the same protocol except for the deuterium exchange step. All reactions were performed in triplicate. Peptide trapping and desalting were carried out using a Waters VanGuard BEH Pre-column 2.1 × 5 mm, and separation was achieved using a Waters BEH C18 reverse-phase column 1.7 µm 1.0 × 50 mm with all liquid chromatography (LC) carried out using a Waters Acquity LC system. Thirty pmol of digested peptides were loaded, trapped, and washed using a 0.1% formic acid solution at 0.1 ml/min, and subsequent separation was carried out using a 14 min 5%–40% acetonitrile gradient at a flow rate of 70 μl/min. Peptide identification was performed by acquiring and processing the MS^E^ data acquired for non-deuterated samples using ProteinLynx Global Server v3.0.1 (PLGS, Waters Corporation). The level of deuterium exchange was examined using HDExaminer software (Sierra Analytics). Significant uptake changes were shown using volcano plots by a confidence threshold of a *p* value of <0.05, and figures were created using VolcaNoseR (Goedhart and Luijsterburg, 2020). The protein structure figures were prepared using PyMOL (The PyMOL Molecular Graphics System, Version 1.7.6.2 Schrödinger, LLC). Protein-DNA interactions from the PDB files were extracted using the DNAproDB database (Sagendorf et al., 2020), and figures were created with BioRender.com.

### Cell culture and cell viability assay

Wild-type (WT) and NEIL1 knockout (KO) Hap1 cell lines were kindly provided by Dr. Magnar Bjørås (Norwegian University of Science and Technology (NTNU), Norway). Cells were cultured in IMDM media supplemented with 10% FBS and 1X penicillin and streptomycin at 37°C and 5% CO_2_. NEIL1 KO cell lines were validated using western blot analysis. WT and NEIL1 KO Hap1 cells from a 10 cm dish were collected by scraping in cell lytic M (Millipore Sigma) and lysed by agitation at 4°C. Cell debris were pelleted by centrifugation at 16,900 × g for 15 min at 4°C and whole cell extract was collected. 50 μg of protein was loaded, separated by SDS-PAGE, and transferred to a PVDF membrane (Biorad). The membrane was probed using a NEIL1 rabbit polyclonal antibody (1:1000; Proteintech #12145-1-AP). PCNA (D3H8P) XP rabbit monoclonal antibody (1:1,000; Cell Signaling #13110S) was used as loading control. An ECL anti-rabbit IgG secondary antibody conjugated to HRP (1:10,000; GE Healthcare NA934V) and WesternBright ECL HRP substrate (Advansta Inc) were used to visualize antibody binding using a BioRad ChemiDoc imager. Cell viability upon methyl methanesulfonate (MMS) treatment was assessed using a resazurin-based fluorescence assay as described previously (D'Arcy et al., 2019). Briefly, cells were seeded (10,000 cells/well) in costar black 96-well clear bottom plates. After 24 h, media containing MMS was added to final concentrations of 1, 3, 10, 30, 100, and 300 μM, 1, 3, 10, and 30 mM by serial dilution. Following 72 h, resazurin solution was added to a final concentration of 120 μM and incubated for 4 h. The relative fluorescence was measured at 540 ± 20 nm excitation and 620 ± 20 nm emission on a Tecan Infinite M1000 Pro multimode plate reader. Non-linear regression analysis [(Inhibitor) vs. response] was performed using GraphPad Prism 8.

### Quantitative real-time PCR and estimation of mtDNA copy number

cDNA was prepared from each cell line, with or without treatment with 125 μM MMS for 72 h, using the TaqMan™ Gene Expression Cells-to-CT™ Kit (#4399002). mRNA expression of four human mitochondrial genes *CYB*, *ND1*, *CO1*, and *RNR1* was determined by quantitative real-time PCR using the TaqMan Fast Universal Master Mix (2X; catalog no. 4352042) and TaqMan Gene Expression Assay probes from Life Technologies (*MT-CYB*, Hs02596867_s1; *MT-ND1*; Hs02596873_s1; *MT-CO1*, Hs02596864_g1; *MT-RNR1*, Hs02596859_g1). The reactions were performed using QuantStudio Pro 7 (Applied Biosystems) RT-PCR system, and analysis of mRNA expression was performed as per the instruction of the manufacturer (∆∆CT method). Transcript quantities were normalized to GAPDH (Hs02758991_g1) as a reference gene transcript.

The absolute and relative mtDNA copy number was estimated for the Hap1 WT and NEIL1 KO cells using Absolute Human Mitochondrial DNA Copy Number Quantification qPCR Assay Kit (ScienCell Research Laboratories #8948) following the manufacturer’s protocol. Briefly, genomic DNA (gDNA) was isolated using GeneJET Genomic DNA Purification Kit (ThermoFisher Scientific #K0721), and 2.5 ng gDNA was used to quantify mtDNA copy number with mtDNA specific primer sets in QuantStudio Pro 7 (Applied Biosystems) RT-PCR system. A reference genomic DNA in the kit was used to calculate the absolute and relative mtDNA copy number. A total of three to four biological replicates were analyzed for these experiments with each experiment performed in triplicate. Statistical analysis was performed using Student’s t-test in GraphPad Prism version 8.1.0 for MAC OS X (GraphPad Software, San Diego, California United States, www.graphpad.com).

## Results

### Mapping the interaction between NEIL1 and TFAM, *in vitro*

**FIGURE 1:**
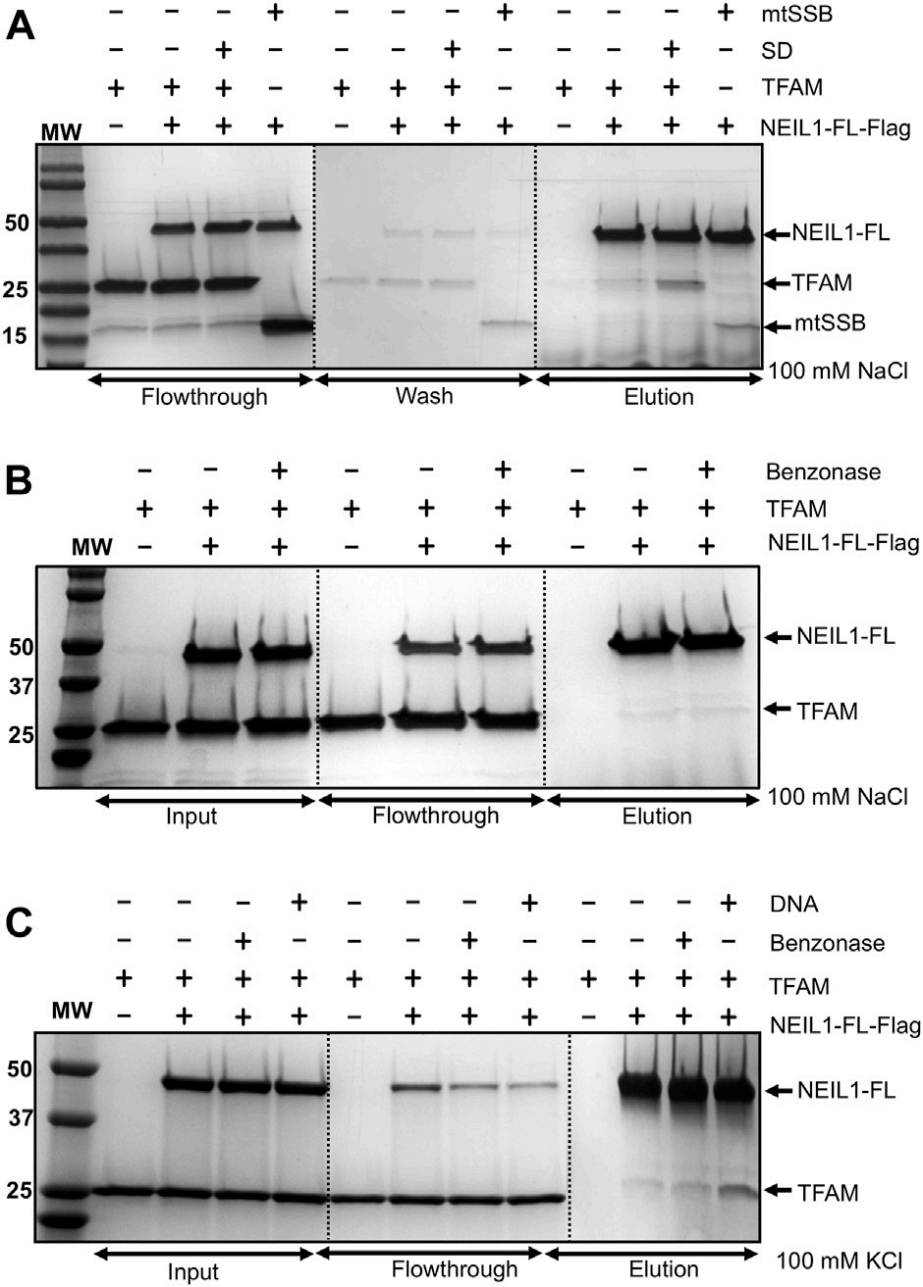
Affinity pull-down experiments reveal an interaction between recombinantly purified NEIL1 and TFAM. **(A)** Flag-tagged, full-length NEIL1 (NEIL1-FL) was used to pull down TFAM in the presence and absence of a specific DNA (SD) sequence containing an abasic site in a buffer containing 100 mM NaCl. TFAM was observed in the elution fractions in both the presence and absence of SD. mtSSB was used as a positive control as we previously documented the interaction between NEIL1 and mtSSB. **(B)** The purified proteins were treated with Benzonase prior to complex formation to eliminate nucleic acid contamination followed by the pull-down experiment. TFAM was observed in elution fractions containing either 100 mM NaCl in the buffer, or 100 mM KCl in the buffer **(C)** in the both Benzonase treated or non-treated samples indicating that there is a direct interaction between the two proteins.

**FIGURE 2:**
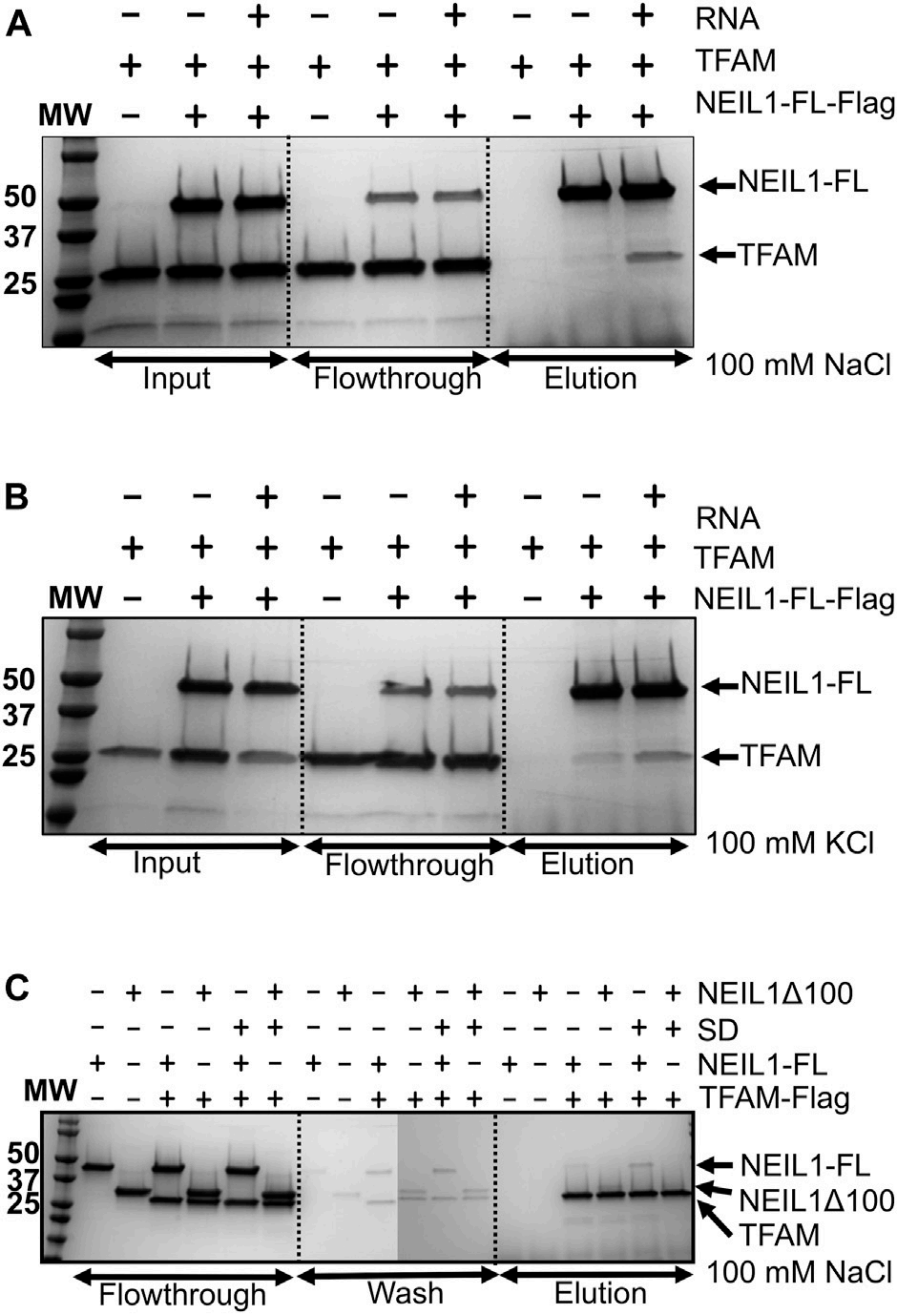
Affinity pull-down experiments display an interaction between NEIL1 and TFAM using recombinantly purified proteins. **(A)** Flag tagged NEIL1 was used to pull down TFAM in the presence of RNA in a buffer containing 100 mM NaCl. TFAM is observed in the elution fractions in the absence and presence of RNA. **(B)** Flag tagged NEIL1 was used to pull down TFAM in the presence of RNA in a buffer containing 100 mM KCl. Under these conditions, TFAM is also observed in the elution fractions in the absence and presence of RNA. **(C)** The reverse pull-down experiment using Flag-tagged TFAM was performed. Full-length NEIL1 (NEIL1-FL) and a truncated NEIL1 enzyme lacking 100 residues from disordered C-terminal region (NEIL1-Δ100) were observed in the elution fractions in the presence and absence of DNA. Non-specific binding of untagged TFAM, NEIL1-FL, and NEIL1-Δ100 with Flag beads was not detected as shown in elution fractions when the interaction partner is absent.

The interaction of NEIL1 with downstream BER factors, as well as with proteins involved with other aspects of DNA metabolism, is essential for the efficient repair of DNA lesions (Hegde et al., 2012). NEIL1’s function has been associated with mitochondrial genome maintenance, as demonstrated by previous work by our group and others (Hu et al., 2005; Vartanian et al., 2006; Sampath et al., 2011; Sharma et al., 2018). In preliminary experiments, using affinity pull-down assays from mammalian cells followed by mass-spectrometry, we identified peptides belonging to interacting partners of NEIL1 including TFAM and mtSSB (data not shown). We previously reported and mapped an interaction between NEIL1 and mtSSB using biochemical and structural approaches (Sharma et al., 2018). In the current manuscript, using recombinantly purified Flag-tagged NEIL1 or TFAM, we show that NEIL1 and TFAM likely interact weakly at concentrations of low salt between 50–100 mM NaCl or 50–150 mM KCl, while a slight interaction was observed at higher salt concentrations (>300 mM NaCl) both in the presence and absence of a 22-mer specific DNA (SD) or RNA substrate containing an abasic site analog (Figures 1A–C, Figures 2A–B; Supplementary Figures S1–S5). We also performed similar experiments in the presence of Benzonase to probe a direct interaction between the two proteins in the absence of any nucleic acid binding partners and noted that the presence of Benzonase did not alter binding between the two proteins (Figures 1B,C). These results indicate that the interaction between NEIL1 and TFAM is most likely transient and can be manipulated by altering the interacting environment where lower salt conditions are favored. Interestingly, we also observed an interaction between TFAM and a truncated polypeptide of NEIL1 that lacks the C-terminal protein interaction domain (called NEIL1Δ100), both in the presence and absence of DNA, indicating that interaction with TFAM is not limited to the residues within the putative protein-binding disordered C-terminal tail of NEIL1 (Figure 2C).

**FIGURE 3:**
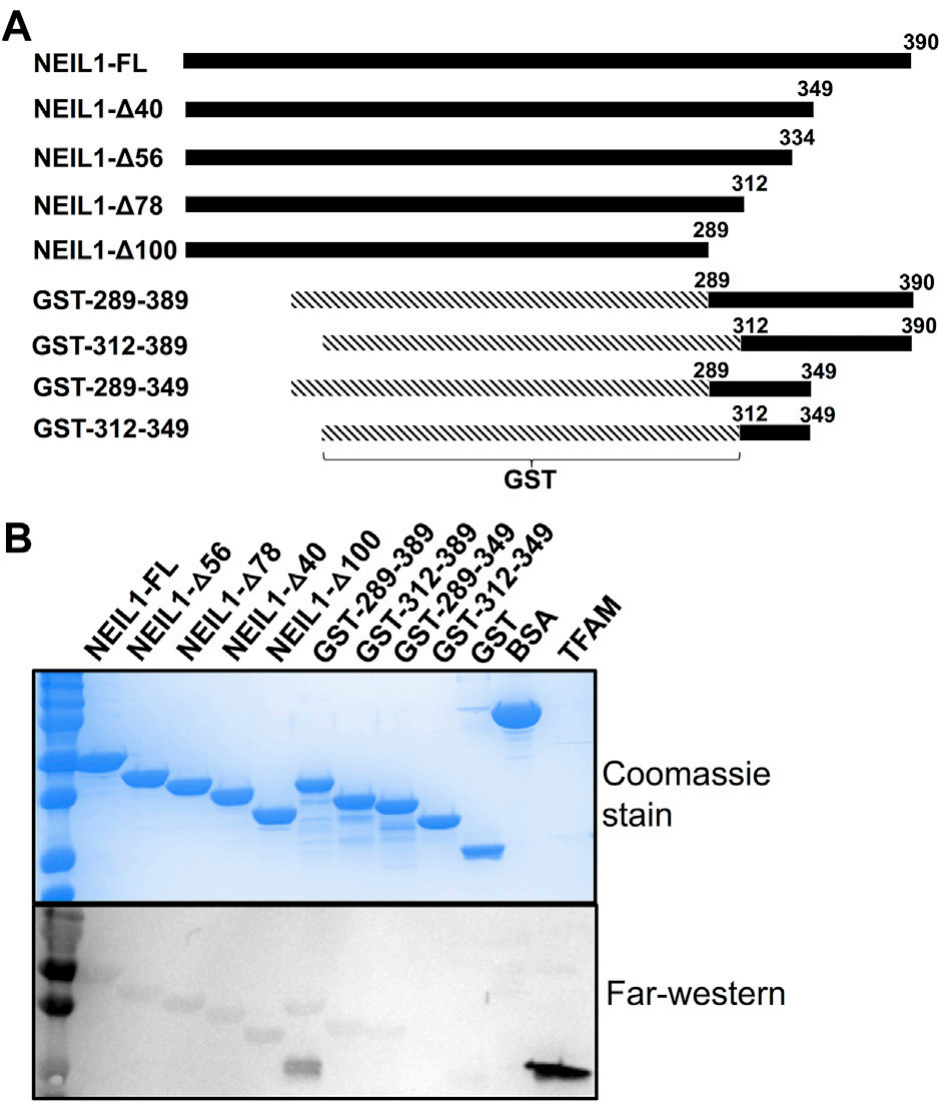
Far-western analysis indicates that NEIL1 interacts with TFAM via multiple binding sites present at both the N- and C-terminal domains. **(A)** A map of the His-tagged polypeptides of NEIL1 lacking portions of the C-terminal disordered tail and the GST-tagged C-terminal polypeptides of NEIL1 lacking the N-terminal portion of the enzyme. **(B)** Far-western analysis to determine the minimal region of NEIL1 required for interaction with TFAM. All proteins used in this study were expressed in *E. coli*, purified to homogeneity, and verified by sodium dodecyl sulfate-polyacrylamide gel electrophoresis (SDS-PAGE) analysis stained with Coomassie blue. 50 pmol of NEIL1 and the truncated enzymes, bovine serum albumin (negative control), glutathione S-transferase (negative control), and 1 pmol of TFAM (positive control) were loaded onto the gel. Far-western analysis was performed where proteins were transferred to a PVDF membrane, denatured, slowly renatured on the membrane, incubated with 10 pmol/ml purified TFAM, and probed with an anti-TFAM antibody to detect an interaction.

To further identify the region of NEIL1 that binds to TFAM, we employed far-western analysis using purified recombinant histidine-tagged full-length NEIL1 (NEIL1-FL) and the truncated polypeptides of NEIL1, with the indicated number of residues deleted: NEIL1-∆40, NEIL1-∆56, NEIL1-∆78, and NEIL1-∆100 (Figures 3A,B top panel). We also expressed and purified GST-tagged NEIL1 polypeptides containing regions of the disordered C-terminus, amino acids 289–390, 289–349, 312–390, and 312–349 (Figures 3A,B top panel) and performed far-western analysis as described previously (Hegde et al., 2012; Prakash et al., 2017). BSA and GST alone were used as negative controls. The membrane with the refolded FL and truncated NEIL1 proteins was incubated with purified TFAM and then probed with an antibody against TFAM. Interestingly, our results indicate that purified TFAM interacts with the N-terminal regions of NEIL1, including the protein that lacks the protein interaction domain (100 residues) (Figure 3B, bottom panel). A slight interaction with TFAM was also observed with the C-terminal polypeptides of NEIL1 except for one that comprised residues 312–349. This indicates that NEIL1 interacts with TFAM *via* multiple binding sites that are present at its N- as well as C-terminal domains, a result that is divergent from past observations with its other binding partners, including mtSSB and the proliferating cell nuclear antigen (PCNA) where NEIL1 interactions were exclusively observed within the disordered C-terminal tail (Prakash et al., 2017; Sharma et al., 2018). Replicates of the far-western analysis are displayed in Supplementary Figure S6.

### Complex formation between NEIL1 and TFAM observed *via* SEC, MALS, and SAXS

From our past experience with recombinant NEIL1, we observed that the protein is unstable and prone to aggregation when subjected to multiple freeze-thaw cycles upon long-term storage. Furthermore, for structural studies involving solution scattering methods where we require milligram amounts of purified protein, we noted that NEIL1 was prone to precipitation at higher concentrations in the presence of a buffer containing lower concentrations of sodium chloride (<100 mM NaCl). Even though complex formation between NEIL1 and TFAM is favored at lower salt conditions, for the SEC studies we used buffer containing 300 mM NaCl to attempt to isolate a protein-protein complex in the presence of DNA. We used a calibrated Superdex 200 column and documented the MW of each eluting species (Supplementary Table S1). We first analyzed the proteins alone to determine their individual retention volumes and calculated the MW values based on a standard curve. NEIL1-FL (theoretical mass of 44.75 kDa) elutes as a single peak with a MW of 49.1 kDa on the SEC column (Supplementary Figure S7A; Supplementary Table S1). The theoretical MW of a full-length TFAM monomer (lacking the N-terminal mitochondrial targeting sequence or MTS) is 25.6 kDa, and on the SEC column TFAM elutes as a single peak with a MW estimate of 39.9 kDa (Supplementary Figure S7A; Supplementary Table S1). The higher MW values calculated from our SEC experiments for the proteins is likely because of their elongated shape that results from the region of disorder within the C-terminal domain of unliganded NEIL1 (residues 290–390) (Doublié et al., 2004; Hegde et al., 2010) and high intrinsic flexibility observed in unliganded TFAM (Rubio-Cosials et al., 2011). When combined, a mixture of NEIL1 and TFAM elutes as two separate peaks at their respective retention volumes, indicating that the two proteins likely do not form a complex that can be isolated under solvent conditions containing 300 mM NaCl (Supplementary Figure S7A; Supplementary Table S1). Next, we performed SEC analysis of the two proteins in the presence of the specific (SD) or non-specific DNA (NSD) substrates. The NEIL1-SD and -NSD complexes elute at a MW value of 52.8 kDa, and the TFAM-DNA complexes elute at ∼45 kDa (45.4 for SD and 43.6 for NSD; Supplementary Figures S7B,C; Supplementary Table S1). The SEC data obtained for the ternary complex (TFAM-NEIL1-DNA) was inconclusive, as we did not observe a clear separate peak at a higher retention volume for the complex (Supplementary Figures S7D–F; Supplementary Table S1). However, given the likely transient nature of these interactions, there may be a small fraction representative of a ternary complex present in the equilibrated mixture of TFAM-NEIL1-DNA, which cannot be deciphered by SEC under our current conditions. We therefore sought to determine the absolute molar mass and the stoichiometry of binding using SEC-MALS-SAXS methods where samples are loaded onto an SEC Superdex 200 column with an exclusion limit of 600 kDa, followed by a UV detector, MALS detector (DAWN Helios II, Wyatt Technologies), and a SAXS flow cell.

SEC-MALS-SAXS data were collected at beamline 18-ID (BioCAT) of the advanced photon source (APS) at Argonne National Laboratory. All measurements were performed in a buffer containing 25 mM HEPES pH 7.4, 5% glycerol, 300 mM NaCl, and 1 mM DTT, which we used previously to scrutinize the NEIL1-mtSSB complex (Sharma et al., 2018). We collected SEC-MALS-SAXS data either in duplicate or triplicate for the proteins individually, protein-DNA complexes (including both SD and NSD), and the protein-protein-DNA ternary complexes where the SEC profiles (top left panels) and SAXS analyses (top right and bottom panels) are displayed in Supplementary Figures S8A–I. The absolute molar mass and MW values calculated by MALS and SAXS are tabulated in Supplementary Table S2. For the ternary complex (TFAM-NEIL1-SD), we collected SEC-SAXS data in triplicate and the data are consistent between two of the three runs. For simplicity, values obtained from run 1 are displayed in Supplementary Table S2. For some of the NEIL1-containing samples, we were able to estimate molar mass values from MALS for only one of the three runs owing to aggregation in solution, as observed in the scattering curves obtained from SAXS analysis (Supplementary Table S2; Supplementary Figures S8A,C). For each sample, we performed evolving factor analysis (EFA) to extract individual components from SEC-SAXS peaks, which revealed multiple overlapping components (*comp*) in solution for some of the samples (Supplementary Table S2). For each scattering species, we observe a distinct profile where the scattering intensity is plotted as a function of momentum transfer (Supplementary Figures S8A–I; Top right Panels). An upward trend at low q-values is observed in samples containing NEIL1 alone and NEIL1-TFAM, which is representative of some aggregation of NEIL1 within the samples. The estimated values of forward scattering intensity [*I* (0)], radius of gyration (*R*_g_), and maximum particle dimension (*D*_max_) from Guinier analysis or pairwise distance distribution, *P*(*r*), analysis are summarized in Supplementary Table S2. *P*(*r*) analysis for both unliganded NEIL1 and TFAM display a curve with an elongated tail that results in large *D*_max_ values of 152 Å and 140 Å, respectively, similar to values we and others reported for these proteins (Rubio-Cosials et al., 2011; Prakash et al., 2017; Rubio-Cosials et al., 2018; Sharma et al., 2018) (Supplementary Figures S8A,B; bottom left panels). The large *D*_max_ values represent a likely elongated shape for these unliganded proteins that result from intrinsic flexibility and the presence of multiple conformations of varying dimensions in solution. This pronounced flexibility is also observed in the Kratky plots for the individual proteins where the plot either does not fully converge to the q axis at high q values or converges at larger q values when compared to the bound proteins described below (Supplementary Figures S8A,B; bottom right panels). When combined, the two proteins NEIL1 and TFAM elute as separate peaks as observed by the SEC profile collected prior to MALS and SAXS, and the *D*_max_ values and Kratky plots are similar to those obtained for the unliganded proteins alone (Supplementary Figure S8C). For the NEIL1-SD/-NSD and TFAM-SD/-NSD complexes, we observe lower *D*_max_ values (126/115 Å for NEIL1-SD/-NSD and 89/95 Å for TFAM-SD/-NSD) in comparison to the unliganded proteins (152 Å for NEIL1; 140 Å for TFAM) suggesting that binding to DNA changes the conformation of the proteins, likely stabilizing them and causing them to be more globular in nature (Supplementary Table S2; Supplementary Figures S8D–G; bottom left panels). Kratky analysis for the proteins bound to DNA display bell-shaped curves characteristic of less flexible, globular molecules (Supplementary Figures S8D–G; bottom right panels). EFA for the ternary complex containing TFAM-NEIL1-SD/-NSD reveals multiple components in solution with *D*_max_ values of 148 Å and 154 Å that best correspond to the TFAM-NEIL1-SD and -NSD complexes, respectively, indicative of a larger linear dimension for the ternary complexes relative to the protein species (Supplementary Table S2; Supplementary Figures S8H,I; bottom left panels). Kratky plots for the ternary complexes are bell-shaped and converge to the q-axis at lower q-values when compared with the proteins alone (Supplementary Figures S8H,I; Bottom right panels).

In summary, corroborating our SEC results, in the absence of DNA, a mixture of NEIL1 and TFAM did not form a complex as indicated by two separate eluting species with MW values corresponding to the individual proteins. However, in the presence of DNA, we observe complex formation as indicated by a peak with a higher molar mass of 91.2 kDa obtained by MALS (Supplementary Table S2. This value could correspond to a ternary complex of TFAM-NEIL1-DNA at either a stoichiometric ratio of 1:1:1 (theoretical MW 83.9 kDa) or 1:1:2 (theoretical MW 97.5 kDa). These results indicate that NEIL1 and TFAM form a ternary complex only in the presence of DNA under our current solvent conditions.

### HDX-MS to identify peptides at the interaction interface

We used HDX-MS, a powerful technique that provides information regarding protein folding, stability, conformational dynamics, and ligand binding, to probe the interaction between NEIL1 and TFAM in the presence of DNA. With HDX-MS we can measure the rate of deuterium uptake when amide hydrogens present in the protein backbone are exposed to deuterated solvent (D_2_O) and exchanged. Well-folded, buried, stable, secondary structural elements of a protein are typically protected from HDX; however, flexible regions and solvent-exposed residues readily take up deuterium. Similarly, upon protein-protein or protein-ligand binding, interaction interfaces are also protected from HDX. To our knowledge, this technique has not been previously used to scrutinize NEIL1, TFAM, or their binding to interacting partners thus presenting a novel methodology to scrutinize these complexes. We therefore systematically analyzed the binding of the specific DNA substrate containing an abasic site analog to NEIL1 and TFAM individually as well as in a complex comprising both proteins. Given the lower concentration of samples required for this technique, we were able to use the buffer containing 100 mM NaCl for these experiments. Both NEIL1 and TFAM bind to double-stranded DNA substrates with nanomolar affinities where Kd’s measured for the NEIL1-DNA complex range from 2–29 nM (Odell et al., 2010; Prakash et al., 2016; Schomacher et al., 2016; Kladova et al., 2019) depending on the lesion and the DNA substrate, and Kd’s measured for TFAM range from 4–7 nM (Gangelhoff et al., 2009; Malarkey et al., 2012; Ngo et al., 2014; Brown et al., 2015; Ramachandran et al., 2017; Cuppari et al., 2019). Prior knowledge of protein-DNA binding and residues involved with the interactions is obtained from published crystal structures of the TFAM-DNA and NEIL1-DNA complexes (Ngo et al., 2014; Zhu et al., 2016). We systematically collected HDX-MS data for the TFAM and NEIL1 proteins individually, the respective protein-DNA complexes, and lastly, the TFAM-NEIL1-DNA complex. For each of our samples, we obtained near-complete peptide coverage where the coverage for the TFAM samples ranged between 84.58%–94.86%, whereas the NEIL1 samples displayed a coverage of 98.74% (Supplementary Figures S9, S10).

**FIGURE 4:**
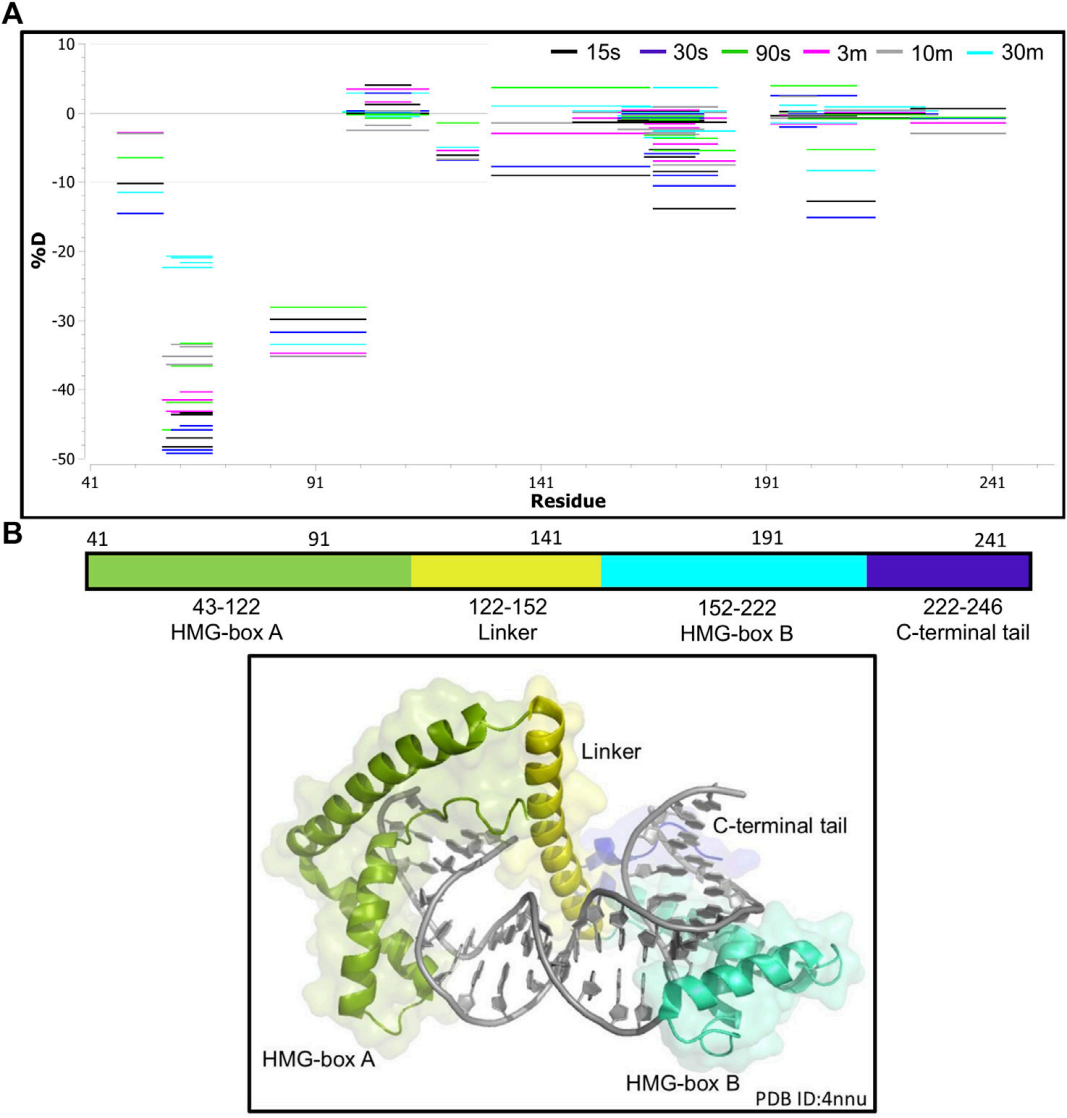
Hydrogen-deuterium exchange of the TFAM-DNA complex reveals the regions of TFAM involved with DNA binding. **(A)** Woods plot representing the distribution of TFAM regions displaying differential levels of solvent protection in the presence of DNA. Percent change in deuteration for peptides after various time points (15 s–30 m) between TFAM and the TFAM-DNA complex, where a negative percentage indicates less deuteration and more protection due to complex formation with the DNA. Each horizontal line in the plot represents an individual peptide with residue range on the *X*-axis and deuteration level i.e., level of protection on the *Y*-axis. **(B)** The domain map and cartoon representation of the crystal structure of the TFAM-DNA complex (PDB ID:4nnu) are displayed. In the structure, the DNA is colored grey and each region is color-matched to the domain map above.

**FIGURE 5:**
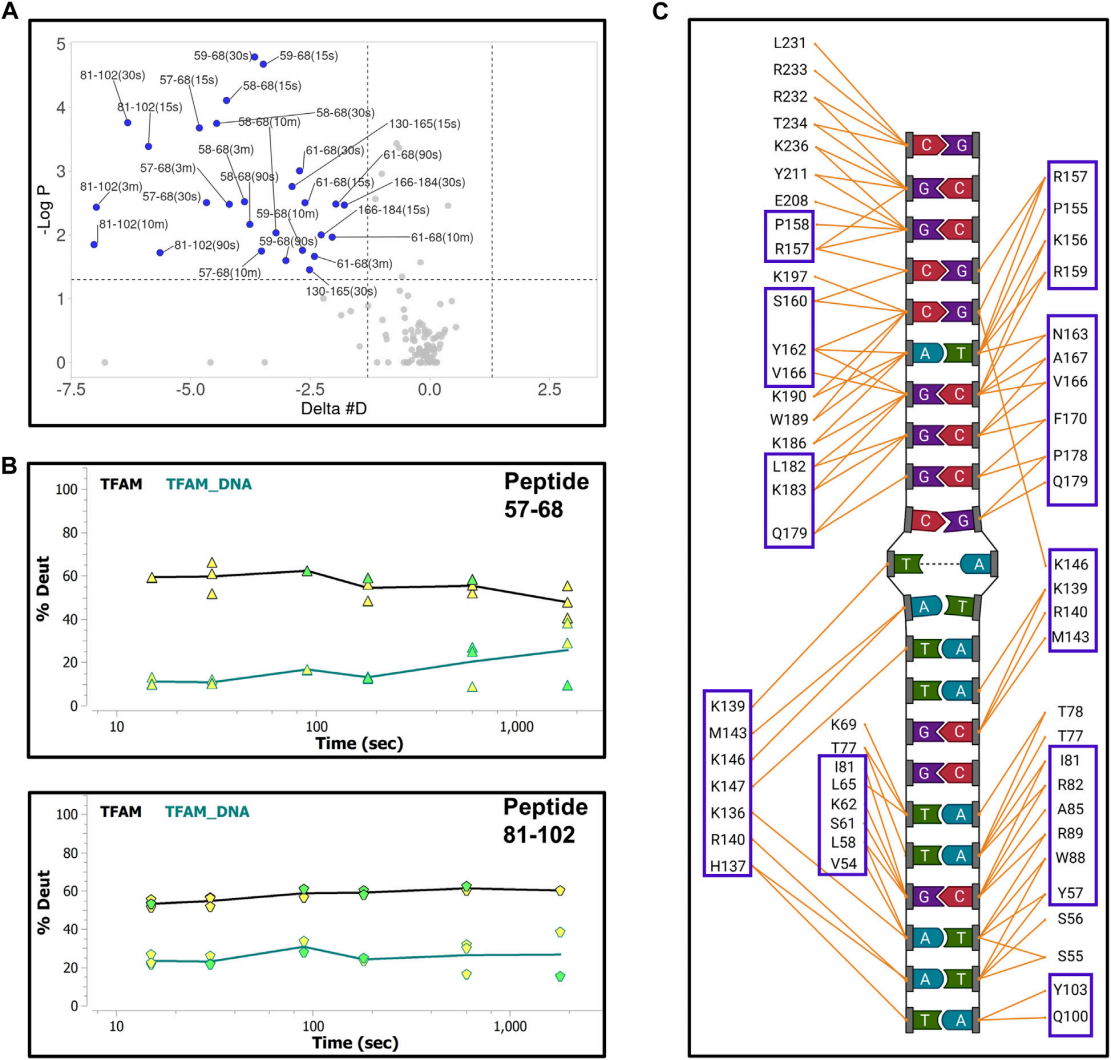
Hydrogen-deuterium exchange of the TFAM-DNA complex reveals the regions of TFAM involved with DNA binding. **(A)** Volcano plot quantifying the significant change in deuteron uptake for each peptide at a given time point. The upper left quadrant of the plot displays peptides (solid blue circles) with a significant decrease in deuteron uptake of the TFAM-DNA complex relative to TFAM alone. This significance is based on two statistical tests performed by the HDExaminer software where the first is a *p*-value test with a significance cutoff value of <0.05 (i.e., at a confidence level of 95%) and the second is based on whether the difference value on the *X*-axis (Delta #D) is greater than the replicate variance across all of the data within each specific data set as determined by the program. **(B)** Representative uptake plots are shown from the HDX-MS time course for peptides 57–68 and 81–102 that are significantly different between the TFAM-DNA and TFAM samples based on the volcano plot in **(A)**. **(C)** Interaction map showing TFAM residues that interact with DNA in the crystal structure of the TFAM-DNA complex (PDB ID:4nnu). Blue boxes represent residues within peptides that display a significant decrease in deuteration observed in the volcano plot.

First, we collected HDX-MS data of the TFAM-DNA complex over varying time points (from 15 s to 30 m), which reveal protection of certain regions within TFAM as indicated by a decrease in deuteration uptake (Woods plot, Figure 4A). We observe a decrease in deuterium exchange for the peptides present in the regions corresponding to high mobility group (HMG) box A (residues 43–122) and HMG-box B (residues 152–222) with an up to 50% decrease for box A and 20% for box B. We noted a ∼10% decrease in deuterium exchange for the peptides within the linker region (residues 122–152) and the C-terminal tail (residues 222–246). These results are consistent with the available structural data for DNA bound TFAM [PDB ID:4nnu, (Ngo et al., 2014)] where the two HMG boxes A and B are mainly involved with DNA binding in addition to some interactions mediated by the linker region (Figure 4B). Four main peptides comprising residues 57–68, 81–102, 130–165, and 166–184 display a significant decrease in deuteration uptake at various time points (*p*-value of <0.05; Figure 5A). Uptake plots for these peptides (Figure 5B) display a consistent decrease in deuteration upon DNA binding. Based on available structural data, these peptides harbor residues L58 and I81 within HMG-box A and N163, P178, and L182 in HMG-box B, which are involved in the intercalation of the DNA minor groove (Ngo et al., 2014). Residues identified from our HDX-MS data that reside within the four peptides (57–68, 81–102, 130–165, and 166–184) are present in the interaction interface of the TFAM-DNA complex (Figure 5C).

**FIGURE 6:**
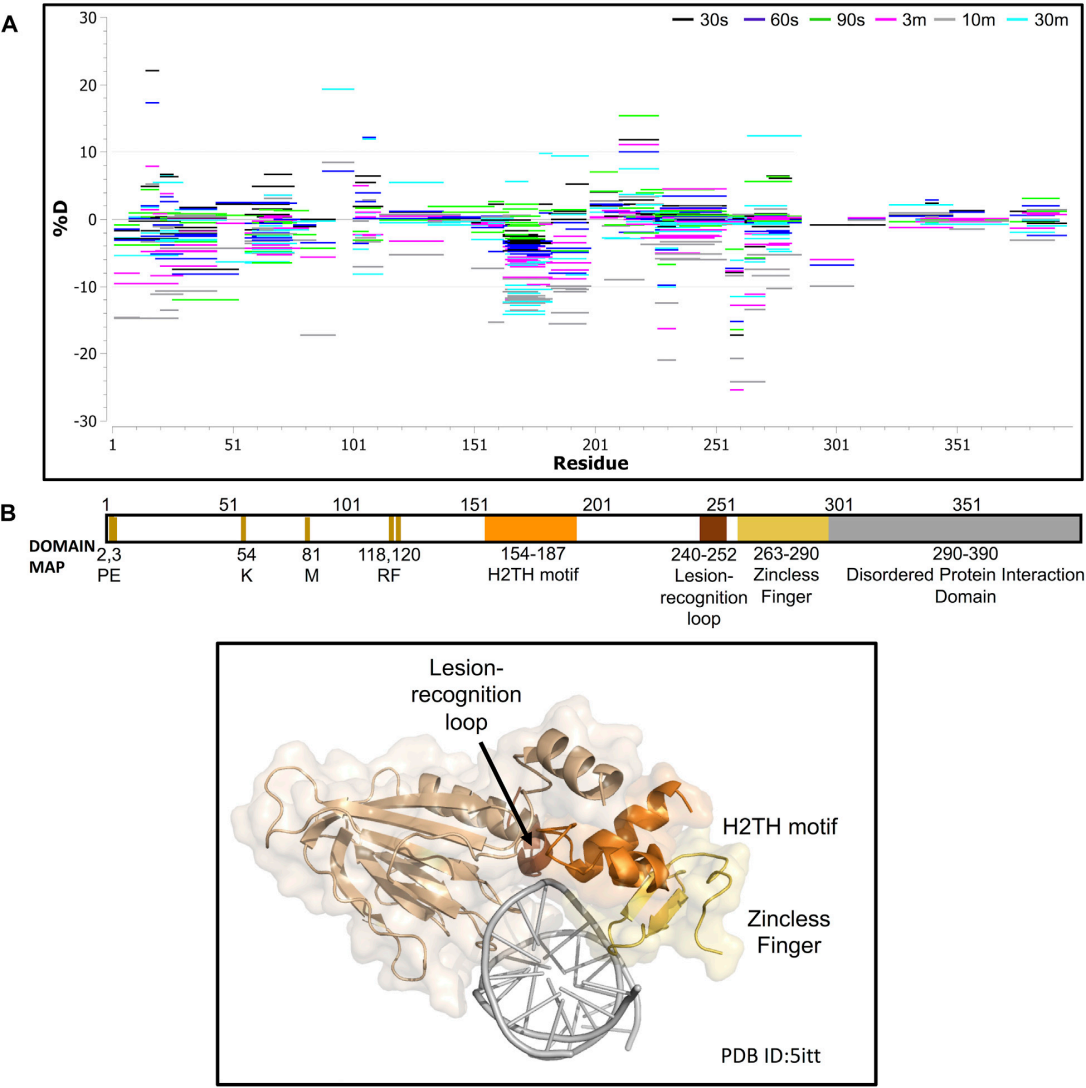
Hydrogen-deuterium exchange experiments for the NEIL1-DNA complex reveals regions of NEIL1 involved with DNA binding. **(A)** Woods plot representing the distribution of NEIL1 regions displaying differential levels of solvent protection in the presence of DNA. Percent change in deuteration for peptides after various time points (30 s–30 m) between NEIL1 and the NEIL1-DNA complex, where a negative percentage indicates less deuteration and more protection as a result of complex formation between NEIL1 and the DNA. Each horizontal line in the plot represents an individual peptide with residue range on the X-axis and deuteration level i.e., level of protection on the *Y*-axis. **(B)** Domain map and cartoon representation of crystal structure of NEIL1-DNA complex (PDB ID:5itt) are displayed. The active site and void-filling residues, and DNA binding motifs are indicated in the domain map. In the structure, the DNA is colored grey, and each region is color-matched to the domain map above.

**FIGURE 7:**
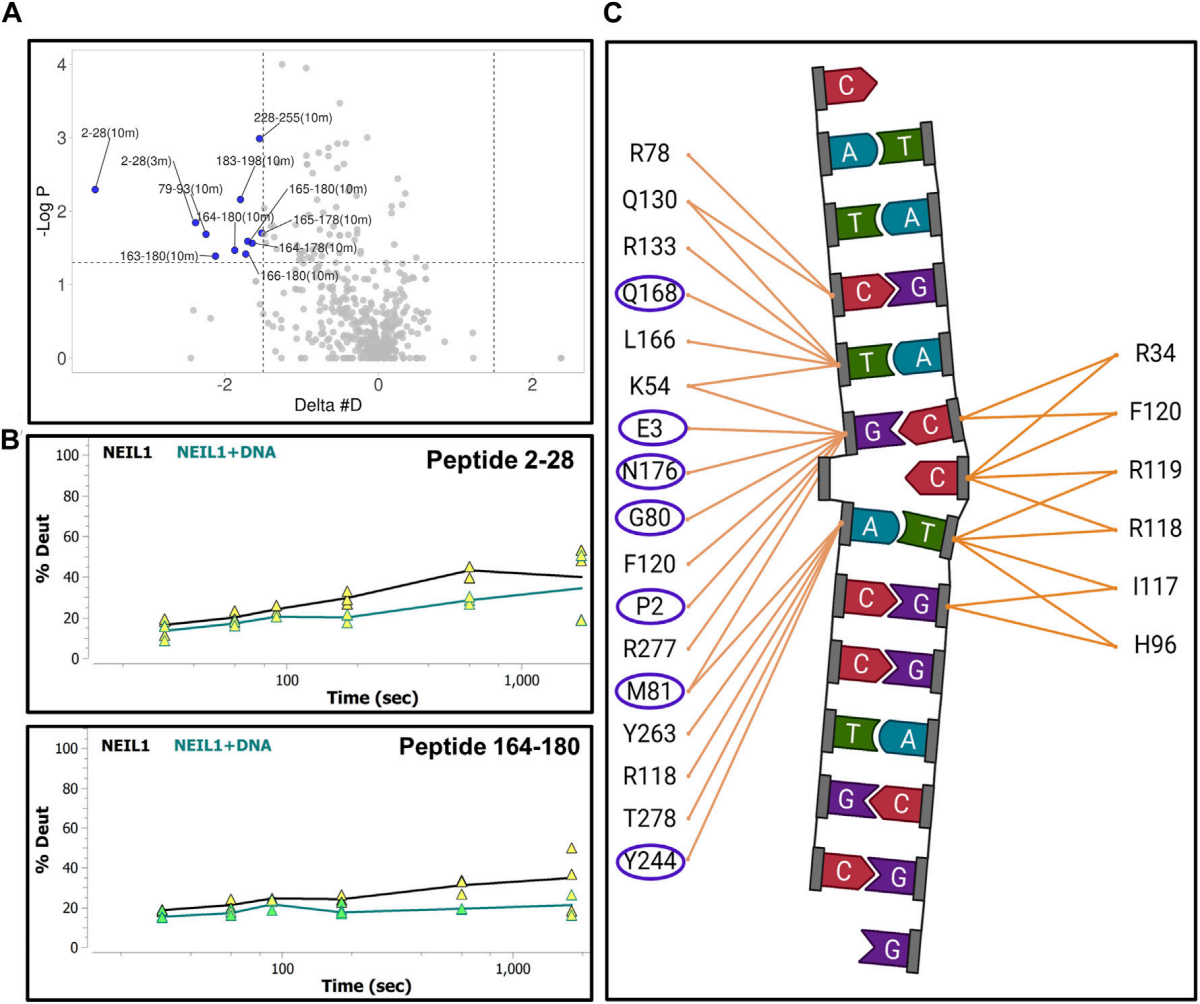
Hydrogen-deuterium exchange experiments for the NEIL1-DNA complex reveals regions of NEIL1 involved with DNA binding. **(A)** Volcano plot quantifying the significant change in deuteron uptake for each peptide at a given time point. The upper left quadrant displays peptides (solid blue circles) at various time points, representing a significant decrease in deuteron uptake upon DNA binding to NEIL1 relative to NEIL1 alone at a *p*-value of <0.05 (please refer to the legend for Figure 5A for a detailed description of the statistical tests used). **(B)** Representative uptake plots are shown from the HDX-MS time course for two of the significant peptides, 2–28 and 164–180, that lie within the significant quadrant in panel **(A)** above. **(C)** Interaction map showing NEIL1 residues that interact with the DNA in the crystal structure of the NEIL1-DNA complex (PDB ID:5itt). The residues within the blue oval circles indicate those present in peptides with a significant decrease in deuteration, as observed in the volcano plot.

We next collected HDX-MS data over a time course (from 30 s to 30 m) for the complex between NEIL1 and DNA, and observed protection at various regions upon DNA binding as indicated by an up to 25% decrease in deuterium uptake (Woods plot; Figure 6A). These regions mainly cluster within the N-terminal domain, harboring the active site residues P2, E3, and K54, and the void-filling residues M81, R118, and F120. We also observed protection at the DNA binding helix-two-turns-helix (H2TH) motif (residues 154–187) and the zincless finger motif (residues 260–293; Figures 6A,B). Peptides that display a significant decrease in deuterium uptake with a confidence *p*-value of <0.05 upon DNA binding lie within regions 2–28, 79–93, 163–180, 183–198, and 228–255 (Volcano analysis, Figure 7A). The uptake plots for these regions show a consistent decrease in deuteration with an additional region identified between residues 256–271, which also display protection upon DNA binding (Figure 7B). The available crystal structures of NEIL1 bound to DNA provide clear confirmation that the peptides that display protection upon DNA binding identified by our HDX-MS data are present within the interaction interface of the NEIL1-DNA complex (Figure 7C).

**FIGURE 8:**
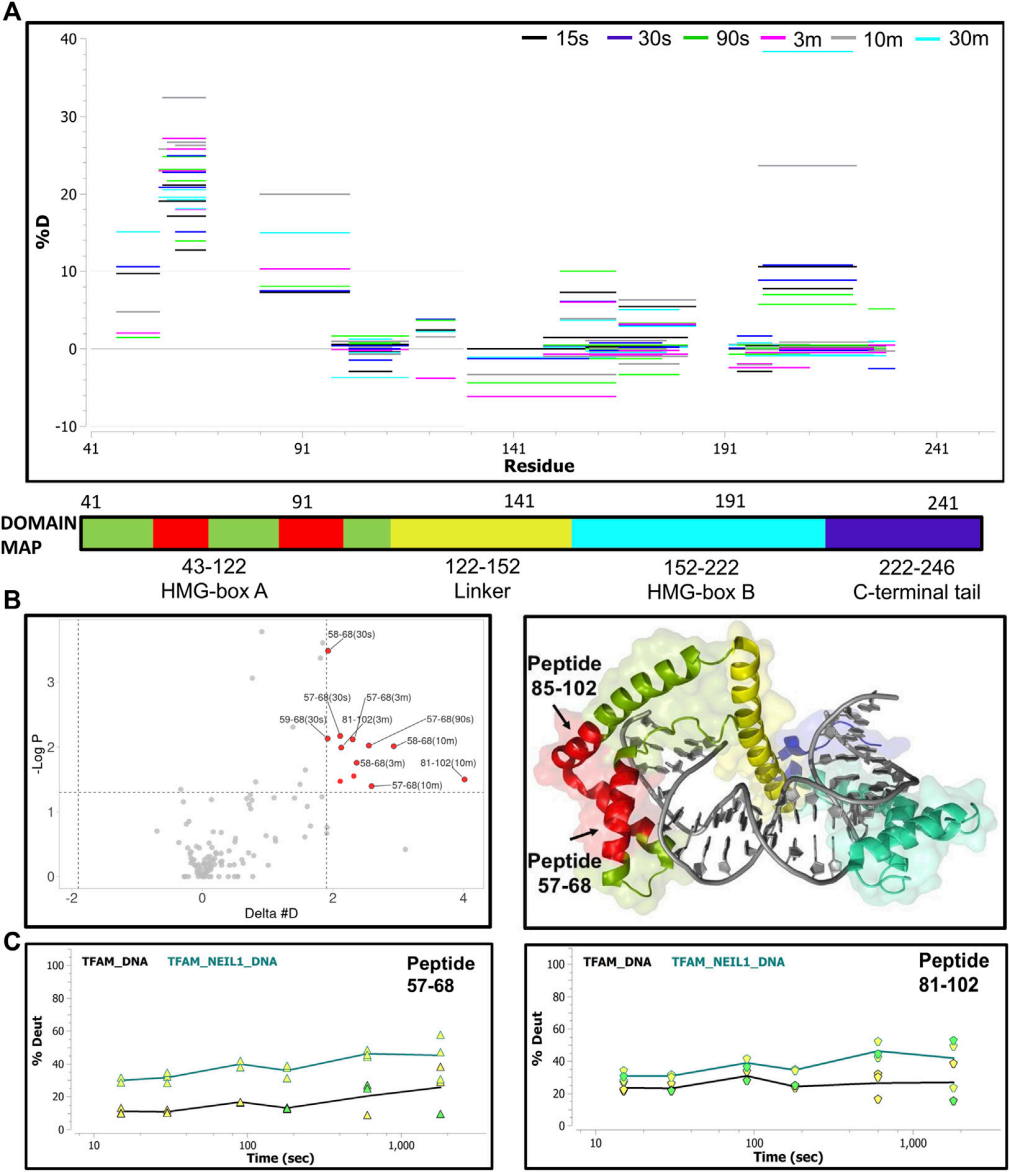
Hydrogen-deuterium exchange analysis of the TFAM-DNA and TFAM-NEIL1-DNA complexes reveals putative TFAM regions that interact with NEIL1 in the presence of DNA. **(A)** Woods plot representing percent change in deuteration for peptides after various time points (15 s–30 m) between the TFAM-DNA and TFAM-NEIL1-DNA complexes, where a positive percentage indicates more deuteration and less protection observed when NEIL1 is present within the TFAM-NEIL1-DNA complex. Each horizontal line in the plot represents an individual peptide with residue range on the *X*-axis and deuteration level i.e., level of protection on the *Y*-axis. **(B)** Volcano plot displaying TFAM peptides with a statistically significant increase in deuteration (*p*-value < 0.05; please refer to the legend for Figure 5A for a detailed description of the statistical tests used) in the TFAM-NEIL1-DNA complex indicated as solid red circles (left panel). On the right panel, the peptides with a significant increase in deuteration are mapped on the crystal structure of the TFAM-DNA complex (PDB ID:4nnu) and are highlighted in red. The domain map above also displays the two regions (red) that show the greatest difference in deuterium uptake upon the addition of NEIL1. **(C)** Representative uptake plots are shown from the HDX-MS time course for peptides 57–68 and 81–102 that lie within the significance quadrant of the volcano plot in **(B)**.

Lastly, we collected HDX-MS data for a sample containing equimolar ratios of TFAM, NEIL1, and DNA at various time points (15 s–30 m) and compared it to the data obtained with the TFAM-DNA sample. Surprisingly, upon the addition of NEIL1, we observed an increase in deuterium uptake for TFAM peptides that are clustered within HMG-box A (with an up to 30% increase) and HMG-box B (with ∼10% increase; Figure 8A). However, only two regions display a significant increase (*p*-value of <0.05) in deuterium uptake, which includes residues 57–68 and 81–102 that are present in HMG-box A (Figure 8B). The peptide uptake plots for the two TFAM regions also show a consistent increase in deuterium exchange when NEIL1 is present in the TFAM-NEIL1-DNA sample (Figure 8C).

**FIGURE 9:**
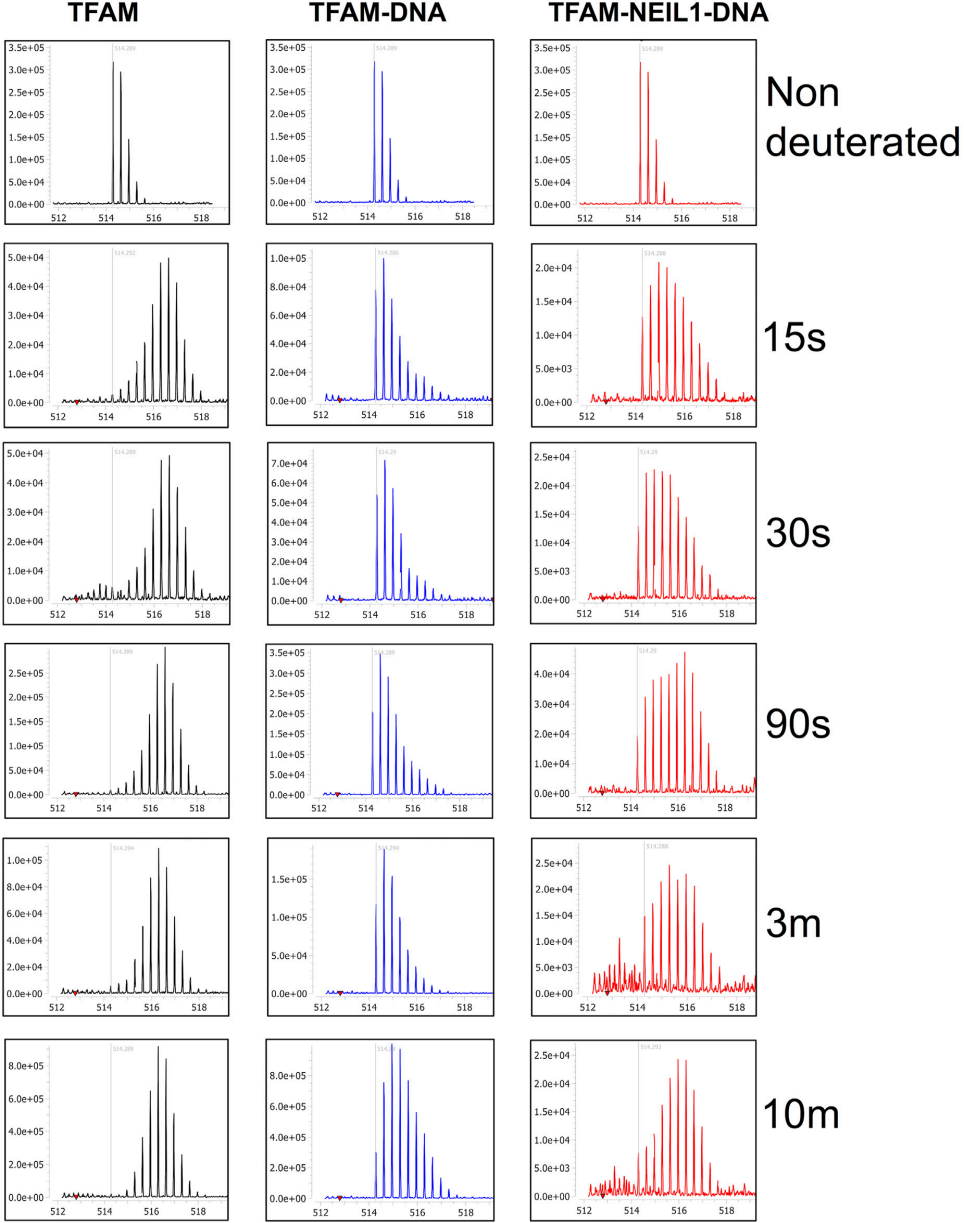
Isotopic mass distribution spectra reveal bimodal deuterium exchange upon the addition of NEIL1 to the TFAM-DNA complex. Isotopic mass distribution spectra from representative HDX-MS experiments for the peptide containing TFAM residues 57–68 at various time points as indicated (15 s–10 m). The distribution pattern for TFAM alone (black) displays greater deuterium exchange when compared to the TFAM-DNA complex (blue), which appears to exchange less deuterium. The addition of NEIL1 to the sample mixture (TFAM-NEIL1-DNA complex; red) reveals a bimodal isotopic mass distribution, which likely results from the presence of multiple species (protein-protein; protein-DNA; protein-protein-DNA; or protein alone) within the sample. The grey line within each plot indicates an *m/z* value of 514.29 corresponding to the non-deuterated peptide.

To probe the origin of the increased exchange upon the addition of NEIL1, we interrogated the isotopic distribution present in the raw data. Protection of TFAM residues 57–68 upon DNA binding is evidenced by comparison of the left and middle panels of Figure 9. In the absence of DNA, the isotopic distribution is shifted towards higher mass (rightward) at early times, whereas, in the presence of DNA the shift is substantially delayed, indicating protection upon DNA binding. When NEIL1 is added to the TFAM-DNA mixture, the isotopic distribution is broadened and appears to be a superposition of the distribution patterns for unbound and DNA-bound TFAM (Figure 9; right panels). This broadened, or bimodal, pattern is indicative of the presence of both protected and unprotected TFAM molecules in solution (Supplementary Figure S11).

### DNA damage by methyl methanesulfonate decreases the transcriptional activity of TFAM in the absence of NEIL1

**FIGURE 10:**
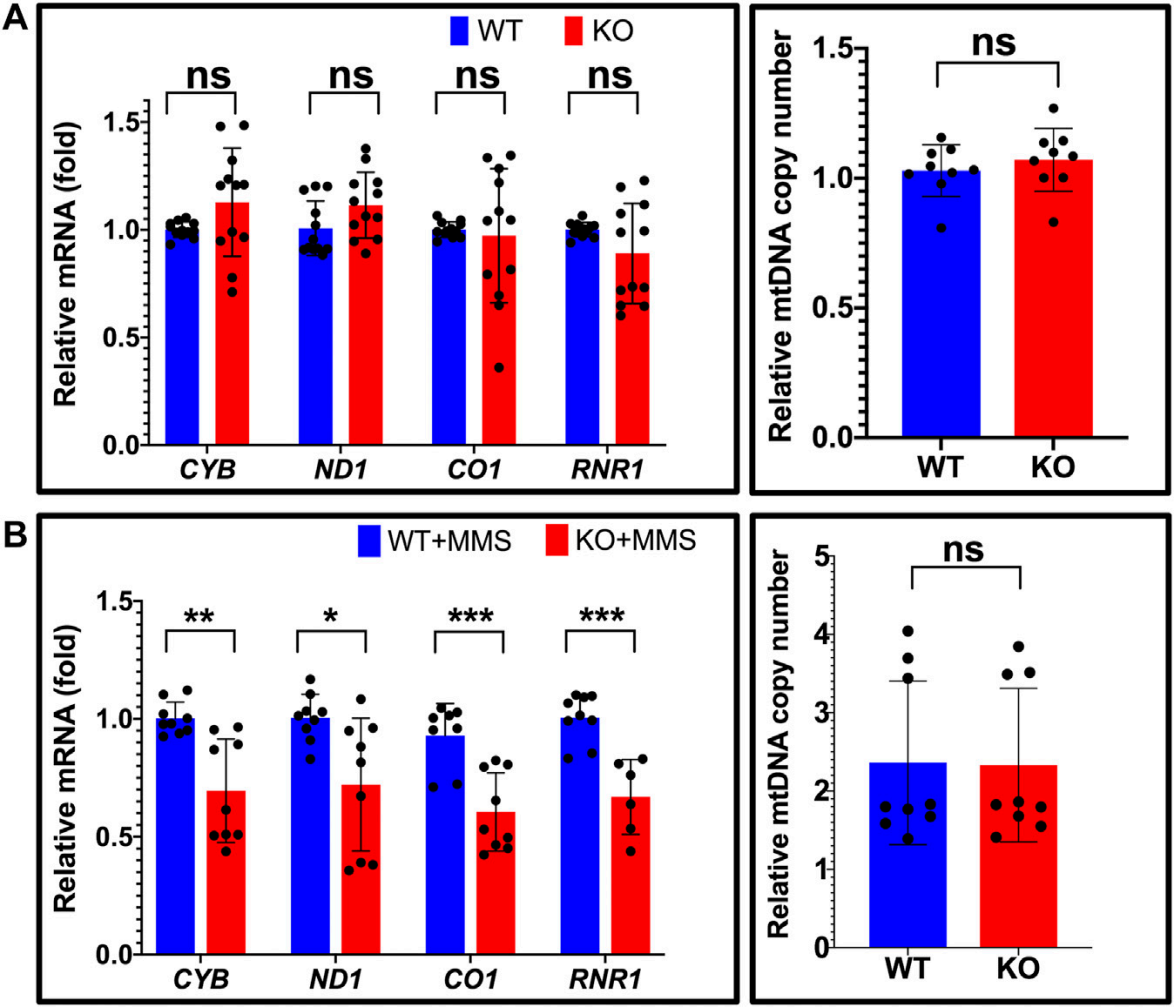
Estimation of relative mitochondrial mRNA expression reveals that NEIL1 is necessary for efficient transcription by TFAM upon DNA damage. **(A)** Left, the relative mRNA expression of four mitochondrial genes encoding Cytochrome b (*CYB*), NADH dehydrogenase subunit 1 (*ND1*), Cytochrome c oxidase I (*CO1*), and 12S ribosomal RNA (*RNR1*) were estimated by qRT-PCR in untreated Hap1 cell lines where the expression of NEIL1 is either intact (i.e., wild-type, WT) or knocked out (i.e., KO). Right, estimation of mitochondrial copy number by qPCR in the WT and KO cell lines. **(B)** Left, the relative mRNA expression of the above four mitochondrial genes in the WT and KO Hap1 cells treated with 125 μM MMS for 3 days prior to gene expression analysis. Right, estimation of mitochondrial copy number by qPCR in the WT and KO cell lines after MMS treatment. Statistical analysis was performed in GraphPad Prism using a Student’s t-test where ns, not significant; *p < 0.05; **p < 0.01; and ***p < 0.001.

To assess the functional impact and the biological role of the interaction between NEIL1 and TFAM, we tested the impact of NEIL1 on the transcriptional activity of TFAM. For these experiments, we used human Hap1 cells where the expression of NEIL1 is either ablated (NEIL1 KO) or unaltered (NEIL1 WT; Supplementary Figure S12A, verified *via* western blotting). Transcriptional activity of TFAM can be monitored by measuring the steady-state mRNA transcript levels of four mitochondrial genes encoding Cytochrome b (*CYB*), NADH dehydrogenase subunit 1 (*ND1*), Cytochrome c oxidase I (*CO1*), and 12S ribosomal RNA (*RNR1*) (Bonekamp et al., 2021). Quantitative reverse transcription PCR (qRT-PCR) analysis for the *CYB*, *ND1*, *CO1*, and *RNR1* genes reveals no significant difference between the WT and NEIL1 KO cells in the absence of any DNA damage indicating that the presence or absence of NEIL1 does not deter the transcriptional activity of TFAM under normal cellular function (Figure 10A, left). To ensure that differences in mtDNA copy number did not influence the qRT-PCR results above, we assessed relative mtDNA copy number and noted no differences between the NEIL1 WT and KO Hap1 cell lines (Figure 10A, right).

Next, we studied the extent to which DNA damage induced by methyl methanesulfonate (MMS) alters the transcriptional activity of TFAM in the presence and absence of NEIL1. We selected the alkylating agent for our studies as the N-glycosyl bond is rendered weak by base alkylation thereby leading to the generation of AP sites, which is a good substrate for both NEIL1 and TFAM (Lindahl, 1993; Friedberg et al., 2005; Vik et al., 2012; Xu et al., 2019). We treated the NEIL1 WT and KO Hap1 cells with an empirically determined concentration (125 μM; Supplementary Figure S12B) of MMS for 3 days and assessed the transcriptional activity of TFAM *via* qRT-PCR as described above. Interestingly, the mRNA expression of all four mitochondrial genes was significantly reduced in the absence of NEIL1 upon MMS treatment (Figure 10B, left), suggesting that NEIL1 is essential for the efficient transcription of mitochondrial genes by TFAM in the presence of DNA damage resulting from MMS. In addition, we did not note any difference in the mtDNA copy number between the NEIL1 WT and KO Hap1 cell lines upon MMS treatment indicating that our results were not altered due to differences in mtDNA copy number (Figure 10B, right).

## Discussion

It is estimated that over 80% of proteins do not function in isolation, but rather work in complexes with other proteins or co-factors to accomplish their roles (Berggard et al., 2007; Rao et al., 2014). As such, the study of protein-protein interactions is essential for understanding cellular processes. These interactions can be either highly stable and permanent or transient and dynamic. While stable interactions are required for macromolecular assemblies like ribosomes to perform their function, transient interactions are important to carry out various signaling and regulatory processes (Acuner Ozbabacan et al., 2011), lending importance to our current endeavor to scrutinize the interaction between NEIL1 and TFAM. While classical biochemical methods used to detect protein-protein interactions can identify robust and stable protein complexes, it is technically challenging to detect interactions between weakly bound, transient protein complexes *in vivo* and *in vitro*. Therefore, choosing appropriate methods that can carefully recognize these dynamic interactions is of the utmost importance. While BER can be thought of as a highly-coordinated, step-wise process involving excision, removal, and restoration of a damaged DNA base, several other factors also mediate the otherwise simplified process. These include and are not limited to protein-protein interactions, post-translational modifications of BER enzymes, and the type of DNA damage (Carter and Parsons, 2016; Moor and Lavrik, 2018). While we and others have provided evidence for the interaction of NEIL1 with several nuclear factors including PCNA, RFC, and RPA (Dou et al., 2008; Theriot et al., 2010; Hegde et al., 2015; Prakash et al., 2017; Sharma et al., 2018), the interaction of NEIL1 with mitochondrial proteins remains underreported. Reports that this enzyme is post-translationally modified by phosphorylation and acetylation have also been described, where we showed that no change in enzyme function was attributed to phosphorylation events *in vitro*, and Mitra and colleagues indicated that acetylation of the enzyme at lysine residues 296–298 is important for nuclear localization and binding to chromatin (Prakash et al., 2016; Sengupta et al., 2018). However, the role of these modifications within the mitochondrion has thus far not been evaluated and requires scrutiny.

**FIGURE 11:**
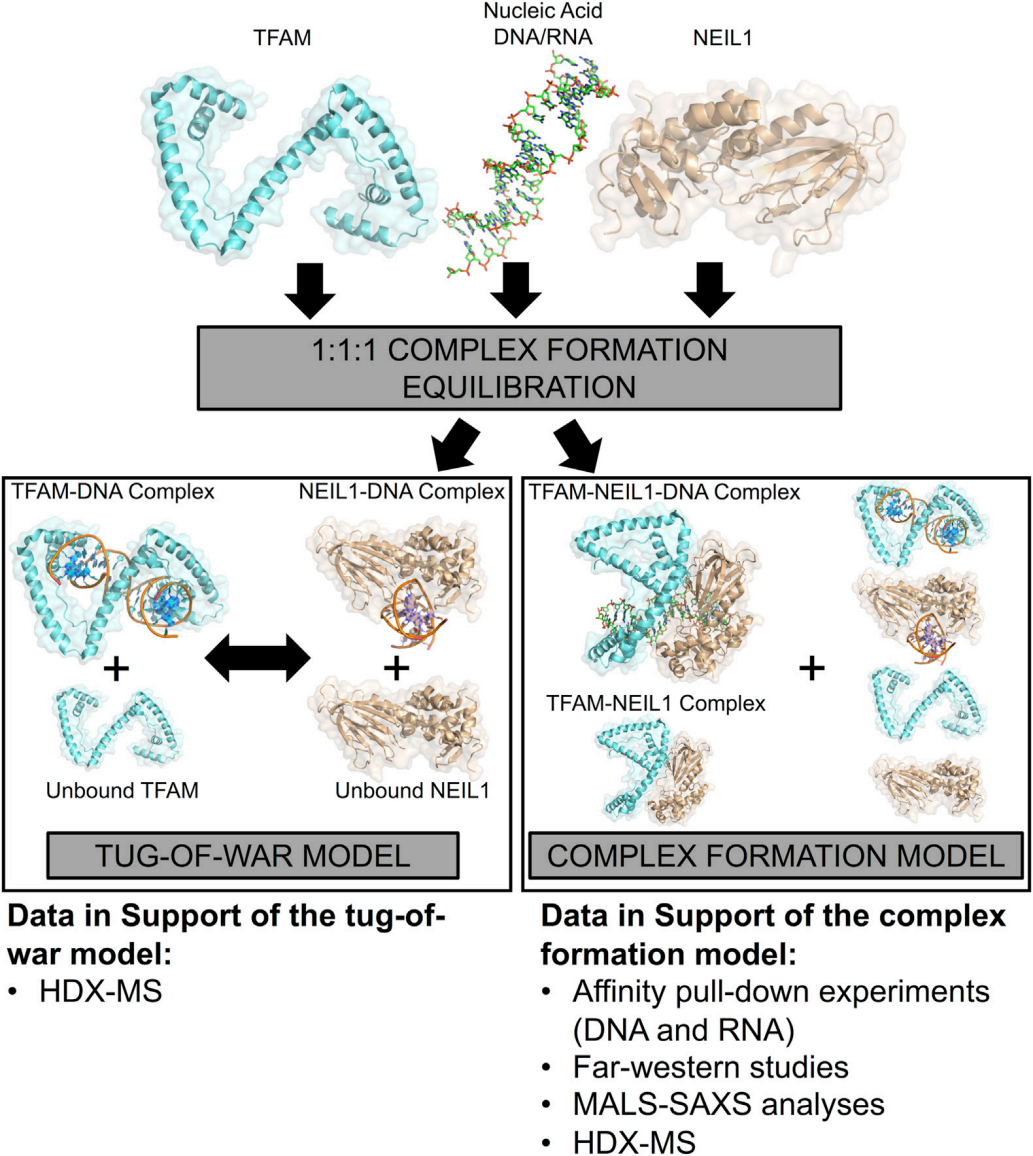
A model representing the interaction between NEIL1 and TFAM in the presence and absence of nucleic acid binding partners. Two scenarios are possible, when NEIL1, TFAM, and DNA are mixed in a 1:1:1 M ratio. In the tug-of-war model, the two proteins compete to form protein-DNA complexes, whereas, in the complex formation model, a small fraction of both proteins interact in the presence and absence of DNA, forming a complex. Species containing protein-DNA complexes or unbound-protein/DNA are also possible in this scenario. The HDX-MS data alone are insufficient to distinguish between the two proposed models but support for the complex formation model is also provided by pull-down, far-western, MALS, and SAXS analyses.

Our efforts to study the interactome of NEIL1 within the context of the mitochondrion have been impeded owing to challenges such as low endogenous cellular levels of NEIL1 and even lower levels of the enzyme within the mitochondrion, as well as the lack of specificity of commercially available antibodies. NEIL1 is typically involved with the recognition and removal of oxidized DNA bases where the frequency at which these lesions occur within the mitochondrion remains to be elucidated; however, the enzyme likely processes oxidized lesions when they occur within mtDNA (Hailer et al., 2005; Krishnamurthy et al., 2008; Albelazi et al., 2019; Han et al., 2019). In this current study, we employed an orthogonal *in vitro* approach to study the interaction between NEIL1 and mitochondrial TFAM in the presence of an abasic site containing DNA duplex that is a favored substrate by both proteins and propose a model for this interaction (Figure 11). In one scenario, when TFAM, NEIL1, and DNA are combined in a 1:1:1 M ratio, we propose that both NEIL1 and TFAM can individually bind to half of the available DNA, leaving some amount of protein unbound. In this tug-of-war model, the two proteins do not interact even in the presence of DNA, but instead compete for the DNA. We also propose a second model, which we refer to as the complex formation model, where a portion of both proteins interact in the presence or absence of DNA forming a complex. Species containing protein-DNA binary complexes or unbound-protein/DNA are also possible in this scenario. To distinguish between these two proposed models, we present data from affinity pull-down experiments, far western studies, SEC-MALS coupled to SAXS, and HDX-MS. Our data indicate that the two proteins appear to interact weakly in the absence of DNA, whereas the presence of DNA favorably alters the interaction landscape. Furthermore, we noted that the interaction can be regulated by changing the buffer conditions, effectively modulating the local binding environment as observed in our affinity pull-down studies where lowering the salt concentration in the buffer favors an interaction. The stoichiometry of the complexes as determined using absolute molar mass values from MALS analysis suggest that larger ternary TFAM-NEIL1-DNA complexes form in the presence of DNA. We further scrutinized the impact of complex formation using HDX-MS, and while this technique does not provide us with atomic resolution structures like NMR, X-ray crystallography, or cryo-electron microscopy, it offers valuable information regarding the conformational dynamics of the protein-DNA binary complexes or protein-protein-DNA ternary complexes studied here (Narang et al., 2020). From our HDX-MS data, it is difficult to distinguish between the tug-of-war model and the complex formation model as the two models present similar species in solution as observed by a bimodal distribution of TFAM peptides upon the addition of NEIL1 within the TFAM-NEIL1-DNA sample; however, evidence from other techniques presented herein favor the complex formation model (Figure 11). We also note that the activity of TFAM is not negatively impacted by the presence (or absence) of NEIL1 under normal cellular conditions. However, we observed aberrant TFAM transcriptional activity upon treatment with MMS in the absence of NEIL1. MMS is a damaging agent which generates primarily 7-methylguanine (7meG) and 3-methlyladenine (3meA) (Beranek, 1990) and while the monofunctional alkyladenine DNA glycosylase (AAG) can excise alkylated bases within the mitochondrion, abasic sites are generated in the process, which are substrates for both NEIL1 and TFAM (van Loon and Samson, 2013; Montaldo et al., 2019). While there is no direct evidence for the involvement of NEIL1 in the repair of 7meG or 3meA, NEIL1 is known to excise other methylated bases such as 2,6-diamino-4-hydroxy-5N-methyl formamidopyrimidine (Fapy-7meG) which could form spontaneously from 7meG in alkaline environments such as that found in the mitochondria (Gates et al., 2004; Prakash et al., 2014). Future work involving mechanistic insight into the interaction between NEIL1 and TFAM in the presence of a panel of oxidative stressors as well as other DNA damaging agents specific to mitochondrial DNA, is warranted.

## Data Availability

The original contributions presented in the study are included in the article/Supplementary Material, further inquiries can be directed to the corresponding author.

## References

[B1] Acuner Ozbabacan S. E. Engin H. B. Gursoy A. Keskin O. (2011). Transient protein-protein interactions. Protein Eng. Des. Sel. 24, 635–648. 10.1093/protein/gzr025 21676899

[B2] Alam T. I. Kanki T. Muta T. Ukaji K. Abe Y. Nakayama H. (2003). Human mitochondrial DNA is packaged with TFAM. Nucleic Acids Res. 31, 1640–1645. 10.1093/nar/gkg251 12626705 PMC152855

[B3] Albelazi M. S. Martin P. R. Mohammed S. Mutti L. Parsons J. L. Elder R. H. (2019). The biochemical role of the human NEIL1 and NEIL3 DNA glycosylases on model DNA replication forks. Genes (Basel) 10, 315. 10.3390/genes10040315 PMC6523847 31018584

[B4] Anderson S. Bankier A. T. Barrell B. G. De Bruijn M. H. Coulson A. R. Drouin J. (1981). Sequence and organization of the human mitochondrial genome. Nature 290, 457–465. 10.1038/290457a0 7219534

[B5] Anson R. M. Hudson E. Bohr V. A. (2000). Mitochondrial endogenous oxidative damage has been overestimated. FASEB J. 14, 355–360. 10.1096/fasebj.14.2.355 10657991

[B6] Baptiste B. A. Baringer S. L. Kulikowicz T. Sommers J. A. Croteau D. L. Brosh R. M. JR. (2021). DNA polymerase beta outperforms DNA polymerase gamma in key mitochondrial base excision repair activities. DNA Repair (Amst) 99, 103050. 10.1016/j.dnarep.2021.103050 33540226 PMC7887074

[B7] Beranek D. T. (1990). Distribution of methyl and ethyl adducts following alkylation with monofunctional alkylating agents. Mutat. Res. 231, 11–30. 10.1016/0027-5107(90)90173-2 2195323

[B8] Berggard T. Linse S. James P. (2007). Methods for the detection and analysis of protein-protein interactions. Proteomics 7, 2833–2842. 10.1002/pmic.200700131 17640003

[B9] Bogenhagen D. F. (2012). Mitochondrial DNA nucleoid structure. Biochim. Biophys. Acta 1819, 914–920. 10.1016/j.bbagrm.2011.11.005 22142616

[B10] Bogenhagen D. F. Rousseau D. Burke S. (2008). The layered structure of human mitochondrial DNA nucleoids. J. Biol. Chem. 283, 3665–3675. 10.1074/jbc.M708444200 18063578

[B11] Bonekamp N. A. Jiang M. Motori E. Garcia Villegas R. Koolmeister C. Atanassov I. (2021). High levels of TFAM repress mammalian mitochondrial DNA transcription *in vivo* . Life Sci. Alliance 4, e202101034. 10.26508/lsa.202101034 34462320 PMC8408345

[B12] Brown T. A. Tkachuk A. N. Clayton D. A. (2015). Mitochondrial transcription factor A (TFAM) binds to RNA containing 4-way junctions and mitochondrial tRNA. PLoS One 10, e0142436. 10.1371/journal.pone.0142436 26545237 PMC4636309

[B13] Campbell C. T. Kolesar J. E. Kaufman B. A. (2012). Mitochondrial transcription factor A regulates mitochondrial transcription initiation, DNA packaging, and genome copy number. Biochim. Biophys. Acta 1819, 921–929. 10.1016/j.bbagrm.2012.03.002 22465614

[B14] Canugovi C. Maynard S. Bayne A. C. Sykora P. Tian J. De Souza-Pinto N. C. (2010). The mitochondrial transcription factor A functions in mitochondrial base excision repair. DNA Repair (Amst) 9, 1080–1089. 10.1016/j.dnarep.2010.07.009 20739229 PMC2955416

[B15] Carter R. J. Parsons J. L. (2016). Base excision repair, a pathway regulated by posttranslational modifications. Mol. Cell. Biol. 36, 1426–1437. 10.1128/MCB.00030-16 26976642 PMC4859697

[B16] Copeland W. C. Longley M. J. (2014). Mitochondrial genome maintenance in health and disease. DNA Repair (Amst) 19, 190–198. 10.1016/j.dnarep.2014.03.010 24780559 PMC4075028

[B17] Cuppari A. Fernandez-Millan P. Battistini F. Tarres-Sole A. Lyonnais S. Iruela G. (2019). DNA specificities modulate the binding of human transcription factor A to mitochondrial DNA control region. Nucleic Acids Res. 47, 6519–6537. 10.1093/nar/gkz406 31114891 PMC6614842

[B18] D'Arcy B. M. Swingle M. R. Papke C. M. Abney K. A. Bouska E. S. Prakash A. (2019). The antitumor drug LB-100 is a catalytic inhibitor of protein phosphatase 2A (PPP2CA) and 5 (PPP5C) coordinating with the active-site catalytic metals in PPP5C. Mol. Cancer Ther. 18, 556–566. 10.1158/1535-7163.MCT-17-1143 30679389 PMC6397705

[B19] Das A. Boldogh I. Lee J. W. Harrigan J. A. Hegde M. L. Piotrowski J. (2007). The human Werner syndrome protein stimulates repair of oxidative DNA base damage by the DNA glycosylase NEIL1. J. Biol. Chem. 282, 26591–26602. 10.1074/jbc.M703343200 17611195

[B20] Dou H. Theriot C. A. Das A. Hegde M. L. Matsumoto Y. Boldogh I. (2008). Interaction of the human DNA glycosylase NEIL1 with proliferating cell nuclear antigen. The potential for replication-associated repair of oxidized bases in mammalian genomes. J. Biol. Chem. 283, 3130–3140. 10.1074/jbc.M709186200 18032376

[B21] Doublié S. Bandaru V. Bond J. P. Wallace S. S. (2004). The crystal structure of human endonuclease VIII-like 1 (NEIL1) reveals a zincless finger motif required for glycosylase activity. Proc. Natl. Acad. Sci. U. S. A. 101, 10284–10289. 10.1073/pnas.0402051101 15232006 PMC478564

[B22] Endo T. Yamano K. Kawano S. (2011). Structural insight into the mitochondrial protein import system. Biochim. Biophys. Acta 1808, 955–970. 10.1016/j.bbamem.2010.07.018 20655871

[B23] Farge G. Falkenberg M. (2019). Organization of DNA in mammalian mitochondria. Int. J. Mol. Sci. 20, 2770. 10.3390/ijms20112770 PMC6600607 31195723

[B24] Franke D. Jeffries C. M. Svergun D. I. (2018). Machine learning methods for X-ray scattering data analysis from biomacromolecular solutions. Biophys. J. 114, 2485–2492. 10.1016/j.bpj.2018.04.018 29874600 PMC6129182

[B25] Friedberg E. C. Walker G. C. Siede W. Wood R. D. (2005). DNA repair and mutagenesis. USA: American Society for Microbiology Press.

[B26] Fromme J. C. Verdine G. L. (2004). Base excision repair. Adv. Protein Chem. 69, 1–41. 10.1016/S0065-3233(04)69001-2 15588838

[B27] Gangelhoff T. A. Mungalachetty P. S. Nix J. C. Churchill M. E. (2009). Structural analysis and DNA binding of the HMG domains of the human mitochondrial transcription factor A. Nucleic Acids Res. 37, 3153–3164. 10.1093/nar/gkp157 19304746 PMC2691818

[B28] Gates K. S. Nooner T. Dutta S. (2004). Biologically relevant chemical reactions of N7-alkylguanine residues in DNA. Chem. Res. Toxicol. 17, 839–856. 10.1021/tx049965c 15257608

[B29] Goedhart J. Luijsterburg M. S. (2020). VolcaNoseR is a web app for creating, exploring, labeling and sharing volcano plots. Sci. Rep. 10, 20560. 10.1038/s41598-020-76603-3 33239692 PMC7689420

[B30] Hailer M. K. Slade P. G. Martin B. D. Rosenquist T. A. Sugden K. D. (2005). Recognition of the oxidized lesions spiroiminodihydantoin and guanidinohydantoin in DNA by the mammalian base excision repair glycosylases NEIL1 and NEIL2. DNA Repair (Amst) 4, 41–50. 10.1016/j.dnarep.2004.07.006 15533836

[B31] Hajizadeh N. R. Franke D. Jeffries C. M. Svergun D. I. (2018). Consensus Bayesian assessment of protein molecular mass from solution X-ray scattering data. Sci. Rep. 8, 7204. 10.1038/s41598-018-25355-2 29739979 PMC5940760

[B32] Han D. Schomacher L. Schule K. M. Mallick M. Musheev M. U. Karaulanov E. (2019). NEIL1 and NEIL2 DNA glycosylases protect neural crest development against mitochondrial oxidative stress. Elife 8, e49044. 10.7554/eLife.49044 31566562 PMC6768664

[B33] Hegde M. L. Hazra T. K. Mitra S. (2010). Functions of disordered regions in mammalian early base excision repair proteins. Cell. Mol. Life Sci. 67, 3573–3587. 10.1007/s00018-010-0485-5 20714778 PMC2996047

[B34] Hegde M. L. Hegde P. M. Arijit D. Boldogh I. Mitra S. (2012). Human DNA glycosylase NEIL1’s interactions with downstream repair proteins is critical for efficient repair of oxidized DNA base damage and enhanced cell survival. Biomolecules 2, 564–578. 10.3390/biom2040564 23926464 PMC3733129

[B35] Hegde M. L. Theriot C. A. Das A. Hegde P. M. Guo Z. Gary R. K. (2008). Physical and functional interaction between human oxidized base-specific DNA glycosylase NEIL1 and flap endonuclease 1. J. Biol. Chem. 283, 27028–27037. 10.1074/jbc.M802712200 18662981 PMC2556012

[B36] Hegde P. M. Dutta A. Sengupta S. Mitra J. Adhikari S. Tomkinson A. E. (2015). The C-terminal domain (ctd) of human DNA glycosylase NEIL1 is required for forming BERosome repair complex with DNA replication proteins at the replicating genome: Dominant negative function of the ctd. J. Biol. Chem. 290, 20919–20933. 10.1074/jbc.M115.642918 26134572 PMC4543652

[B37] Hopkins J. B. Gillilan R. E. Skou S. (2017). BioXTAS RAW: Improvements to a free open-source program for small-angle X-ray scattering data reduction and analysis. J. Appl. Crystallogr. 50, 1545–1553. 10.1107/S1600576717011438 29021737 PMC5627684

[B38] Hu J. De Souza-Pinto N. C. Haraguchi K. Hogue B. A. Jaruga P. Greenberg M. M. (2005). Repair of formamidopyrimidines in DNA involves different glycosylases: Role of the OGG1, NTH1, and NEIL1 enzymes. J. Biol. Chem. 280, 40544–40551. 10.1074/jbc.M508772200 16221681

[B39] Kaufman B. A. Durisic N. Mativetsky J. M. Costantino S. Hancock M. A. Grutter P. (2007). The mitochondrial transcription factor TFAM coordinates the assembly of multiple DNA molecules into nucleoid-like structures. Mol. Biol. Cell 18, 3225–3236. 10.1091/mbc.e07-05-0404 17581862 PMC1951767

[B40] Kauppila J. H. Stewart J. B. (2015). Mitochondrial DNA: Radically free of free-radical driven mutations. Biochim. Biophys. Acta 1847, 1354–1361. 10.1016/j.bbabio.2015.06.001 26050972

[B41] Kladova O. A. Grin I. R. Fedorova O. S. Kuznetsov N. A. Zharkov D. O. (2019). Conformational dynamics of damage processing by human DNA glycosylase NEIL1. J. Mol. Biol. 431, 1098–1112. 10.1016/j.jmb.2019.01.030 30716333

[B42] Konarev P. V. Volkov V. V. Sokolova A. V. Koch M. H. Svergun D. I. (2003). Primus: A windows PC-based system for small-angle scattering data analysis. J. Appl. Crystallogr. 36, 1277–1282. 10.1107/s0021889803012779

[B43] Krishnamurthy N. Zhao X. Burrows C. J. David S. S. (2008). Superior removal of hydantoin lesions relative to other oxidized bases by the human DNA glycosylase hNEIL1. Biochemistry 47, 7137–7146. 10.1021/bi800160s 18543945 PMC2574819

[B44] Krokan H. E. Bjoras M. (2013). Base excision repair. Cold Spring Harb. Perspect. Biol. 5, a012583. 10.1101/cshperspect.a012583 23545420 PMC3683898

[B45] Kukat C. Davies K. M. Wurm C. A. Spahr H. Bonekamp N. A. Kuhl I. (2015). Cross-strand binding of TFAM to a single mtDNA molecule forms the mitochondrial nucleoid. Proc. Natl. Acad. Sci. U. S. A. 112, 11288–11293. 10.1073/pnas.1512131112 26305956 PMC4568684

[B46] Kukat C. Wurm C. A. Spahr H. Falkenberg M. Larsson N. G. Jakobs S. (2011). Super-resolution microscopy reveals that mammalian mitochondrial nucleoids have a uniform size and frequently contain a single copy of mtDNA. Proc. Natl. Acad. Sci. U. S. A. 108, 13534–13539. 10.1073/pnas.1109263108 21808029 PMC3158146

[B47] Lim K. S. Jeyaseelan K. Whiteman M. Jenner A. Halliwell B. (2005). Oxidative damage in mitochondrial DNA is not extensive. Ann. N. Y. Acad. Sci. 1042, 210–220. 10.1196/annals.1338.023 15965065

[B48] Lindahl T. (1993). Instability and decay of the primary structure of DNA. nature 362, 709–715. 10.1038/362709a0 8469282

[B49] Liu M. Zhang J. Zhu C. Zhang X. Xiao W. Yan Y. (2021). DNA repair glycosylase hNEIL1 triages damaged bases via competing interaction modes. Nat. Commun. 12, 4108. 10.1038/s41467-021-24431-y 34226550 PMC8257757

[B50] Malarkey C. S. Bestwick M. Kuhlwilm J. E. Shadel G. S. Churchill M. E. (2012). Transcriptional activation by mitochondrial transcription factor A involves preferential distortion of promoter DNA. Nucleic Acids Res. 40, 614–624. 10.1093/nar/gkr787 21948790 PMC3258160

[B51] Meisburger S. P. Taylor A. B. Khan C. A. Zhang S. Fitzpatrick P. F. Ando N. (2016). Domain movements upon activation of phenylalanine hydroxylase characterized by crystallography and chromatography-coupled small-angle X-ray scattering. J. Am. Chem. Soc. 138, 6506–6516. 10.1021/jacs.6b01563 27145334 PMC4896396

[B52] Minko I. G. Vartanian V. L. Tozaki N. N. Coskun E. Coskun S. H. Jaruga P. (2020). Recognition of DNA adducts by edited and unedited forms of DNA glycosylase NEIL1. DNA Repair (Amst) 85, 102741. 10.1016/j.dnarep.2019.102741 31733589 PMC7069121

[B53] Mishmar D. Levin R. Naeem M. M. Sondheimer N. (2019). Higher order organization of the mtDNA: Beyond mitochondrial transcription factor A. Front. Genet. 10, 1285. 10.3389/fgene.2019.01285 31998357 PMC6961661

[B54] Montaldo N. P. Bordin D. L. Brambilla A. Rosinger M. Fordyce Martin S. L. Bjoras K. O. (2019). Alkyladenine DNA glycosylase associates with transcription elongation to coordinate DNA repair with gene expression. Nat. Commun. 10, 5460. 10.1038/s41467-019-13394-w 31784530 PMC6884549

[B55] Moor N. A. Lavrik O. I. (2018). Protein-protein interactions in DNA base excision repair. Biochemistry. 83, 411–422. 10.1134/S0006297918040120 29626928

[B56] Narang D. Lento C. D J. W. (2020). HDX-MS: An analytical tool to capture protein motion in action. Biomedicines 8, 224. 10.3390/biomedicines8070224 PMC7399943 32709043

[B57] Ngo H. B. Kaiser J. T. Chan D. C. (2011). The mitochondrial transcription and packaging factor Tfam imposes a U-turn on mitochondrial DNA. Nat. Struct. Mol. Biol. 18, 1290–1296. 10.1038/nsmb.2159 22037171 PMC3210390

[B58] Ngo H. B. Lovely G. A. Phillips R. Chan D. C. (2014). Distinct structural features of TFAM drive mitochondrial DNA packaging versus transcriptional activation. Nat. Commun. 5, 3077. 10.1038/ncomms4077 24435062 PMC3936014

[B59] Odell I. D. Newick K. Heintz N. H. Wallace S. S. Pederson D. S. (2010). Non-specific DNA binding interferes with the efficient excision of oxidative lesions from chromatin by the human DNA glycosylase, NEIL1. DNA Repair (Amst) 9, 134–143. 10.1016/j.dnarep.2009.11.005 20005182 PMC2829949

[B60] Piiadov V. Ares De Araujo E. Oliveira Neto M. Craievich A. F. Polikarpov I. (2019). SAXSMoW 2.0: Online calculator of the molecular weight of proteins in dilute solution from experimental SAXS data measured on a relative scale. Protein Sci. 28, 454–463. 10.1002/pro.3528 30371978 PMC6319763

[B61] Prakash A. Cao V. B. Doublie S. (2016). Phosphorylation sites identified in the NEIL1 DNA glycosylase are potential targets for the JNK1 kinase. PLoS One 11, e0157860. 10.1371/journal.pone.0157860 27518429 PMC4982613

[B62] Prakash A. Carroll B. L. Sweasy J. B. Wallace S. S. Doublie S. (2014). Genome and cancer single nucleotide polymorphisms of the human NEIL1 DNA glycosylase: Activity, structure, and the effect of editing. DNA Repair (Amst) 14, 17–26. 10.1016/j.dnarep.2013.12.003 24382305 PMC3926126

[B63] Prakash A. Doublié S. (2015). Base excision repair in the mitochondria. J. Cell. Biochem. 116, 1490–1499. 10.1002/jcb.25103 25754732 PMC4546830

[B64] Prakash A. Doublie S. Wallace S. S. (2012). The fpg/nei family of DNA glycosylases: Substrates, structures, and search for damage. Prog. Mol. Biol. Transl. Sci. 110, 71–91. 10.1016/B978-0-12-387665-2.00004-3 22749143 PMC4101889

[B65] Prakash A. Moharana K. Wallace S. S. Doublié S. (2017). Destabilization of the PCNA trimer mediated by its interaction with the NEIL1 DNA glycosylase. Nucleic Acids Res. 45, 2897–2909. 10.1093/nar/gkw1282 27994037 PMC5389659

[B66] Rahman S. Copeland W. C. (2019). POLG-related disorders and their neurological manifestations. Nat. Rev. Neurol. 15, 40–52. 10.1038/s41582-018-0101-0 30451971 PMC8796686

[B67] Ramachandran A. Basu U. Sultana S. Nandakumar D. Patel S. S. (2017). Human mitochondrial transcription factors TFAM and TFB2M work synergistically in promoter melting during transcription initiation. Nucleic Acids Res. 45, 861–874. 10.1093/nar/gkw1157 27903899 PMC5314767

[B68] Rambo R. P. Tainer J. A. (2013). Accurate assessment of mass, models and resolution by small-angle scattering. Nature 496, 477–481. 10.1038/nature12070 23619693 PMC3714217

[B69] Rao V. S. Srinivas K. Sujini G. N. Kumar G. N. (2014). Protein-protein interaction detection: Methods and analysis. Int. J. Proteomics 2014, 147648. 10.1155/2014/147648 24693427 PMC3947875

[B70] Ruan L. Wang Y. Zhang X. Tomaszewski A. Mcnamara J. T. Li R. (2020). Mitochondria-associated proteostasis. Annu. Rev. Biophys. 49, 41–67. 10.1146/annurev-biophys-121219-081604 31928428

[B71] Rubio-Cosials A. Battistini F. Gansen A. Cuppari A. Bernado P. Orozco M. (2018). Protein flexibility and synergy of HMG domains underlie U-turn bending of DNA by TFAM in solution. Biophys. J. 114, 2386–2396. 10.1016/j.bpj.2017.11.3743 29248151 PMC6028807

[B72] Rubio-Cosials A. Sidow J. F. Jimenez-Menendez N. Fernandez-Millan P. Montoya J. Jacobs H. T. (2011). Human mitochondrial transcription factor A induces a U-turn structure in the light strand promoter. Nat. Struct. Mol. Biol. 18, 1281–1289. 10.1038/nsmb.2160 22037172

[B73] Sagendorf J. M. Markarian N. Berman H. M. Rohs R. (2020). DNAproDB: An expanded database and web-based tool for structural analysis of DNA-protein complexes. Nucleic Acids Res. 48, D277–D287. 10.1093/nar/gkz889 31612957 PMC7145614

[B74] Saki M. Prakash A. (2017). DNA damage related crosstalk between the nucleus and mitochondria. Free Radic. Biol. Med. 107, 216–227. 10.1016/j.freeradbiomed.2016.11.050 27915046 PMC5449269

[B75] Sampath H. Batra A. K. Vartanian V. Carmical J. R. Prusak D. King I. B. (2011). Variable penetrance of metabolic phenotypes and development of high-fat diet-induced adiposity in NEIL1-deficient mice. Am. J. Physiol. Endocrinol. Metab. 300, E724–E734. 10.1152/ajpendo.00387.2010 21285402 PMC3074946

[B76] Schomacher L. Han D. Musheev M. U. Arab K. Kienhofer S. Von Seggern A. (2016). Neil DNA glycosylases promote substrate turnover by Tdg during DNA demethylation. Nat. Struct. Mol. Biol. 23, 116–124. 10.1038/nsmb.3151 26751644 PMC4894546

[B77] Sengupta S. Yang C. Hegde M. L. Hegde P. M. Mitra J. Pandey A. (2018). Acetylation of oxidized base repair-initiating NEIL1 DNA glycosylase required for chromatin-bound repair complex formation in the human genome increases cellular resistance to oxidative stress. DNA Repair (Amst) 66-67, 1–10. 10.1016/j.dnarep.2018.04.001 29698889 PMC5992913

[B78] Sharma N. Chakravarthy S. Longley M. J. Copeland W. C. Prakash A. (2018). The C-terminal tail of the NEIL1 DNA glycosylase interacts with the human mitochondrial single-stranded DNA binding protein. DNA Repair (Amst) 65, 11–19. 10.1016/j.dnarep.2018.02.012 29522991 PMC5911420

[B79] Svergun D. (1992). Determination of the regularization parameter in indirect-transform methods using perceptual criteria. J. Appl. Crystallogr. 25, 495–503. 10.1107/s0021889892001663

[B80] Szczesny B. Tann A. W. Longley M. J. Copeland W. C. Mitra S. (2008). Long patch base excision repair in mammalian mitochondrial genomes. J. Biol. Chem. 283, 26349–26356. 10.1074/jbc.M803491200 18635552 PMC2546560

[B81] Theriot C. A. Hegde M. L. Hazra T. K. Mitra S. (2010). RPA physically interacts with the human DNA glycosylase NEIL1 to regulate excision of oxidative DNA base damage in primer-template structures. DNA Repair (Amst) 9, 643–652. 10.1016/j.dnarep.2010.02.014 20338831 PMC2883689

[B82] Van Houten B. Hunter S. E. Meyer J. N. (2016). Mitochondrial DNA damage induced autophagy, cell death, and disease. Front. Biosci. 21, 42–54. 10.2741/4375 PMC4750375 26709760

[B83] van Loon B. Samson L. D. (2013). Alkyladenine DNA glycosylase (AAG) localizes to mitochondria and interacts with mitochondrial single-stranded binding protein (mtSSB). DNA Repair (Amst) 12, 177–187. 10.1016/j.dnarep.2012.11.009 23290262 PMC3998512

[B84] Vartanian V. Lowell B. Minko I. G. Wood T. G. Ceci J. D. George S. (2006). The metabolic syndrome resulting from a knockout of the NEIL1 DNA glycosylase. Proc. Natl. Acad. Sci. U. S. A. 103, 1864–1869. 10.1073/pnas.0507444103 16446448 PMC1413631

[B85] Vik E. S. Alseth I. Forsbring M. Helle I. H. Morland I. Luna L. (2012). Biochemical mapping of human NEIL1 DNA glycosylase and AP lyase activities. DNA Repair (Amst) 11, 766–773. 10.1016/j.dnarep.2012.07.002 22858590

[B86] Wallace S. S. Murphy D. L. Sweasy J. B. (2012). Base excision repair and cancer. Cancer Lett. 327, 73–89. 10.1016/j.canlet.2011.12.038 22252118 PMC3361536

[B87] Wiedemann N. Pfanner N. (2017). Mitochondrial machineries for protein import and assembly. Annu. Rev. Biochem. 86, 685–714. 10.1146/annurev-biochem-060815-014352 28301740

[B88] Xu W. Boyd R. M. Tree M. O. Samkari F. Zhao L. (2019). Mitochondrial transcription factor A promotes DNA strand cleavage at abasic sites. Proc. Natl. Acad. Sci. U. S. A. 116, 17792–17799. 10.1073/pnas.1911252116 31413200 PMC6731678

[B89] Yakes F. M. Van Houten B. (1997). Mitochondrial DNA damage is more extensive and persists longer than nuclear DNA damage in human cells following oxidative stress. Proc. Natl. Acad. Sci. U. S. A. 94, 514–519. 10.1073/pnas.94.2.514 9012815 PMC19544

[B90] Yeo J. Lotsof E. R. Anderson-Steele B. M. David S. S. (2021). RNA editing of the human DNA glycosylase NEIL1 alters its removal of 5-hydroxyuracil lesions in DNA. Biochemistry 60, 1485–1497. 10.1021/acs.biochem.1c00062 33929180 PMC10294587

[B91] Zhao X. Krishnamurthy N. Burrows C. J. David S. S. (2010). Mutation versus repair: NEIL1 removal of hydantoin lesions in single-stranded, bulge, bubble, and duplex DNA contexts. Biochemistry 49, 1658–1666. 10.1021/bi901852q 20099873 PMC2872175

[B92] Zhu C. Lu L. Zhang J. Yue Z. Song J. Zong S. (2016). Tautomerization-dependent recognition and excision of oxidation damage in base-excision DNA repair. Proc. Natl. Acad. Sci. U. S. A. 113, 7792–7797. 10.1073/pnas.1604591113 27354518 PMC4948311

