# Supplementary material for: Novel interaction interfaces mediate the interaction between the NEIL1 DNA glycosylase and mitochondrial transcription factor A
Source: Front Cell Dev Biol. 2022 Jul 22;10:893806. doi: 10.3389/fcell.2022.893806 (PMC9354671; doi:10.3389/fcell.2022.893806)

**Supplementary Material**

# Novel interaction interfaces mediate the interaction between the NEIL1 DNA Glycosylase and Mitochondrial Transcription Factor A

Nidhi Sharma[1], Marlo K. Thompson[1], Jennifer F. Arrington[1], Dava M.Terry[1], Srinivas Chakravarthy[2], Peter E. Prevelige[3], and Aishwarya Prakash[1]*

[1]University of South Alabama, Department of Biochemistry and Molecular Biology, Mitchell Cancer Institute. 1660 Springhill Avenue, Mobile, AL - 36604.

[2]Illinois Institute of Technology, Advanced photon source, Bldg. 435B/ Sector 18, 9700 S. Cass Avenue, Argonne, IL 60439-4860

[3]University of Alabama, at Birmingham, Department of Microbiology, 845 19th St. South Birmingham AL. 35294-2170

* To whom correspondence should be addressed.

Tel.: 251-410-4915

E-mail: aprakash@southalabama.edu

**Key words:** Base Excision Repair, NEIL1 DNA glycosylase, mitochondrial transcription factor A, size exclusion chromatography, small angle X-ray scattering, HDX-MS

**Running title:** Characterization of the NEIL1-TFAM complex

**CONTENT:**

**I. Supplementary Tables**
**II. Supplementary Legends and Figures**

## I. Supplementary Tables

### Supplementary Table 1: Molecular Weight (MW) values determined by Size Exclusion Chromatography (SEC) using a Superdex 200 3.2/300 column

| | *NEIL1* | *TFAM* | *NEIL1-TFAM* | | *NEIL1-SD** | | *NEIL1-NSD** | | *TFAM-SD** | | *TFAM-NSD** | | *NEIL1-TFAM-SD** | | | *NEIL1-TFAM-NSD** | | |
|---|---|---|---|---|---|---|---|---|---|---|---|---|---|---|---|---|---|---|
| | | | *Peak 1* | *Peak 2* | *Peak 1* | *Peak 2* | *Peak 1* | *Peak 2* | *Peak 1* | *Peak 2* | *Peak 1* | *Peak 2* | *Peak 1* | *Peak 2* | *Peak 3* | *Peak 1* | *Peak 2* | *Peak 3* |
| **Calculated MW** | 49.1 | 39.9 | 51.0 | 41.8 | 52.8 | 34.4 | 52.8 | 34.4 | 45.4 | 36.3 | 43.6 | 34.4 | 51.0 | 41.8 | 34.4 | 51.0 | 41.8 | 34.4 |

* double-stranded DNA substrates used in these studies is described in Supplementary Figure 1 where the X represents either thymine in the non-specific DNA substrate (NSD) or tetrahydrofuran, an abasic site analog, in the specific substrate (SD)

## Supplementary Table 2: SAXS and MALS data collection parameters and calculated molecular weight values

| **(A) Sample details** | | | | | | | | | |
|---|---|---|---|---|---|---|---|---|---|
| | ***NEIL1*** | ***TFAM*** | ***NEIL1-TFAM*** | ***NEIL1-SD**** | ***NEIL1-NSD**** | ***TFAM-SD**** | ***TFAM-NSD**** | ***NEIL1-TFAM-SD**** | ***NEIL1-TFAM-NSD**** |
| Organism | *Homo sapiens* | *Homo sapiens* | *Homo sapiens* | *Homo sapiens* | *Homo sapiens* | *Homo sapiens* | *Homo sapiens* | *Homo sapiens* | *Homo sapiens* |
| Source | *E. coli* | *E. coli* | *E. coli* | *E. coli* | *E. coli* | *E. coli* | *E. coli* | *E. coli* | *E. coli* |
| UniProt ID #Residues in constructs | Q96FI4 (1-390) | Q00059 (43-246) | | | | | | | |
| Extinction coeff. [A280, 0.1%(w/v)] | 0.725 | 1.38 | | | | | | | |
| SEC–SAXS-MALS column, Superdex 200 Increase 10/300 | | | | | | | | | |
| Loading concentration (uM ) | 190 | 190 | 190 | 190 | 190 | 190 | 190 | 190 | 190 |
| Injection volume (µl) | 300 | 300 | 300 | 300 | 300 | 300 | 300 | 300 | 300 |
| Flow rate (ml $min^{-1}$) | 0.7 | 0.7 | 0.7 | 0.7 | 0.7 | 0.7 | 0.7 | 0.7 | 0.7 |
| Buffer | 25 mM HEPES pH 7.4, 5% glycerol, 300 mM NaCl, and 1 mM DTT | | | | | | | | |
| **(B) Data collection parameters** | | | | | | | | | |
| Beamline | APS | APS | APS | APS | APS | APS | APS | APS | APS |
| Wavelength (Å) | 1.03 | 1.03 | 1.03 | 1.03 | 1.03 | 1.03 | 1.03 | 1.03 | 1.03 |
| *Q* Range ($Å^{-1}$) | 0.0043-0.35 | 0.0043-0.35 | 0.0043-0.35 | 0.0043-0.35 | 0.0043-0.35 | 0.0043-0.35 | 0.0043-0.35 | 0.0043-0.35 | 0.0043-0.35 |
| Temperature (°C) | 25 | 25 | 25 | 25 | 25 | 25 | 25 | 25 | 25 |
| **(C) Software used for data reduction, analysis and interpretation** | | | | | | | | | |
| SAXS data reduction | BioXTAS Raw version 2.1.0 | | | | | | | | |
| SAXS data analysis | BioXTAS Raw (EFA), ATSAS | | | | | | | | |
| Extinction coeff. estimate | ProtParam (Expasy) | | | | | | | | |

| **(D) Structural parameters** | | | | | | | | | | | | | | | | | | | | |
|---|---|---|---|---|---|---|---|---|---|---|---|---|---|---|---|---|---|---|---|---|
| | ***NEIL1*** | | ***TFAM*** | ***NEIL1-TFAM*** | | ***NEIL1-SD**** | | ***NEIL1-NSD**** | | | ***TFAM-SD**** | | ***TFAM-NSD**** | | ***TFAM-NEIL1-SD**** | | | ***TFAM-NEIL1-NSD**** | | |
| | ***Comp 1*** | ***Comp 2*** | ***Comp 1*** | ***Comp 1*** | ***Comp 2*** | ***Comp 1*** | ***Comp 2*** | ***Comp 1*** | ***Comp 2*** | ***Comp 3*** | ***Comp 1*** | ***Comp 2*** | ***Comp 1*** | ***Comp 2*** | ***Comp 1*** | ***Comp 2*** | ***Comp 3*** | ***Comp 1*** | ***Comp 2*** | ***Comp 3*** |
| *I*(0) from *P*(r) | 0.07 +/- 1.87e-3 | 0.18 +/- 8.49e-4 | 2.88e-4 +/- 3.83e-6 | 0.07 +/- 5.75e-4 | 0.03 +/- 3.69e-4 | 0.44 +/- 5.52e-4 | 0.02 +/- 2.48e-4 | 0.05 +/- 3.93e-4 | 0.27 +/- 4.09e-4 | 0.01 +/- 2.09e-4 | 0.15 +/- 2.27e-4 | 0.01 +/- 1.58e-4 | 0.18 +/- 2.64e-4 | 0.02 +/- 2.66e-4 | 0.02 +/- 7.16e-4 | 0.36 +/- 1.17e-3 | 0.12 +/- 3.32e-4 | 0.33 +/- 6.51e-4 | 0.19 +/- 5.21e-4 | nd |
| $R_g$ (Å) from *P*(r) | 53.85 +/- 2.23 | 36.25 +/- 0.36 | 33.83 +/- 0.93 | 40.26 +/- 0.78 | 30.69 +/- 0.69 | 31.87 +/- 0.09 | 24.39 +/- 0.27 | 41.92 +/- 0.38 | 31.53 +/- 0.11 | 27.83 +/- 0.57 | 26.77 +/- 0.06 | 24.07 +/- 0.36 | 28.33 +/- 0.06 | 25.1 +/- 0.65 | 61.51 +/- 1.38 | 38.5 +/- 0.24 | 26.32 +/- 0.12 | 40.78 +/- 0.15 | 29.61 +/- 0.1 | nd |
| *I*(0) from Guinier | 0.07 +/- 9.08e-4 | 0.17 +/- 4.87e-4 | 2.87e-4 +/- 2.81e-6 | 0.07 +/- 4.27e-4 | 0.03 +/- 3.61e-4 | 0.44 +/- 4.85e-4 | 0.02 +/- 3.1e-4 | 0.05 +/- 4.62e-4 | 0.26 +/- 3.87e-4 | 0.01 +/- 2.26e-4 | 0.15 +/- 1.99e-4 | 0.01 +/- 1.71e-4 | 0.18 +/- 2.69e-4 | 0.02 +/- 2.78e-4 | 0.02 +/- 1.04e-3 | 0.35 +/- 9.99e-4 | 0.12 +/- 3.69e-4 | 0.32 +/- 6.37e-4 | 0.19 +/- 5.12e-4 | nd |
| $R_g$ from Guinier | 44.5 +/- 0.84 | 32.35 +/- 0.14 | 31.87 +/- 0.55 | 35.22 +/- 0.38 | 27.33 +/- 0.68 | 31.03 +/- 0.07 | 23.74 +/- 0.55 | 41.7 +/- 0.64 | 30.76 +/- 0.08 | 25.49 +/- 0.91 | 26.55 +/- 0.06 | 23.11 +/- 0.54 | 28.1 +/- 0.08 | 25.42 +/- 0.76 | 42.57 +/- 3.99 | 37.08 +/- 0.18 | 25.75 +/- 0.15 | 39.16 +/- 0.13 | 29.25 +/- 0.14 | nd |
| Dmax (Å) | 220 | 152 | 140 | 171 | 112 | 126 | 68 | 127 | 115 | 90 | 89 | 73 | 95 | 96 | 203 | 148 | 87 | 154 | 94 | nd |
| **(E) Molecular weight determination (kDa)** | | | | | | | | | | | | | | | | | | | | |
| Expected Theoretical (Expasy) | 44.7 | | 25.6 | 70.3 | | 58.3 | | 58.3 | | | 39.2 | | 39.2 | | 83.9 | | | 83.9 | | |
| $MW(V_p)$ | 84.1 | 56.3 | 40.2 | 63.3 | 17.6 | nd | nd | 158.9 | 66.4 | 28.2 | 40.3 | 10.3 | 50.9 | nd | 231.9 | 107 | 45.8 | 119.9 | 63.2 | nd |
| $MW(V_c)$ | 55.9 | 44.6 | 34.2 | 47.7 | 17.3 | nd | nd | 136.1 | 57.2 | 30.4 | 38.6 | 19 | 47.2 | nd | 452.7 | 89.2 | 43.3 | 98.7 | 57.7 | nd |
| MW(S&S) | - | 53.3 | 45 | 55.9 | 28.5 | nd | nd | 152.5 | 69.6 | 34 | 47.4 | 22.5 | 53.1 | nd | nd | 102.9 | 47.8 | 111.8 | 58.9 | nd |
| MW (Bayes) | 62.4 | 49.8 | 36.9 | 53.1 | 16.8 | nd | nd | 138.2 | 58.1 | 28.9 | 37.7 | 14.2 | 47.7 | nd | 104.9 | 94.2 | 43.8 | 101 | 58.1 | nd |
| MW (MALS) | nd | nd | 26.1 | 57.5 | 29.1 | 56.3 | nd | 78.1 | 57.2 | nd | 34.3 | nd | 41.2 | nd | 91.2** | 68.7 | 51.6 | nd | 79 | 52.7 |

***** double-stranded DNA substrates used in these studies is described in Supplementary Figure 1 where the X represents either thymine in the non-specific DNA substrate (NSD) or tetrahydrofuran, an abasic site analog, in the specific substrate (SD).
"*Comp"* refers to different components obtained during EFA. While all the data for each component is reported in the table above, the highlighted data (light orange) corresponding to the most well-behaved species as determined by scattering curves and Kratky analysis are discussed in the main manuscript.
**The peaks used to calculate the MW values using MALS and SAXS do not necessarily correspond to the same species. The values that match best with our theoretical understanding of the complex are highlighted in light purple and further discussed.

## II. Supplementary Figures

**Supplementary Figure 1:** Sequences of the duplex DNA and RNA substrates used in our studies. In the non-specific DNA substrate (NSD), the X refers to a thymine and in the specific DNA (SD) sequence the X represents an abasic site analog, tetrahydrofuran (THF).

```
5′- ATTCAACCAAXAGCCCTGGCCG- 3′  Non-Specific DNA (NSD) X=T
3′- TAAGTTGGTTATCGGGACCGGC- 5′  Specific DNA (SD) X=THF
```

```
5′- rArUrUrCrArArCrCrArAXrArGrCrCrCrUrGrGrCrCrG- 3′
                  X=THF
```

**Supplementary Figure 2:** Affinity pull-down experiments reveal an interaction between NEIL1 and TFAM under conditions of low salt between 50-100 mM NaCl. **(A)** Recombinantly expressed Flag-tagged NEIL1 was used to pull down TFAM in the presence and absence of a specific DNA (SD) sequence containing an abasic site under conditions of 50 mM NaCl. **(B-C)** Replicate gel analyses of the affinity pull-down experiments displayed in Figure 1 of the main manuscript using Flag-tagged NEIL1 at 100 mM NaCl.

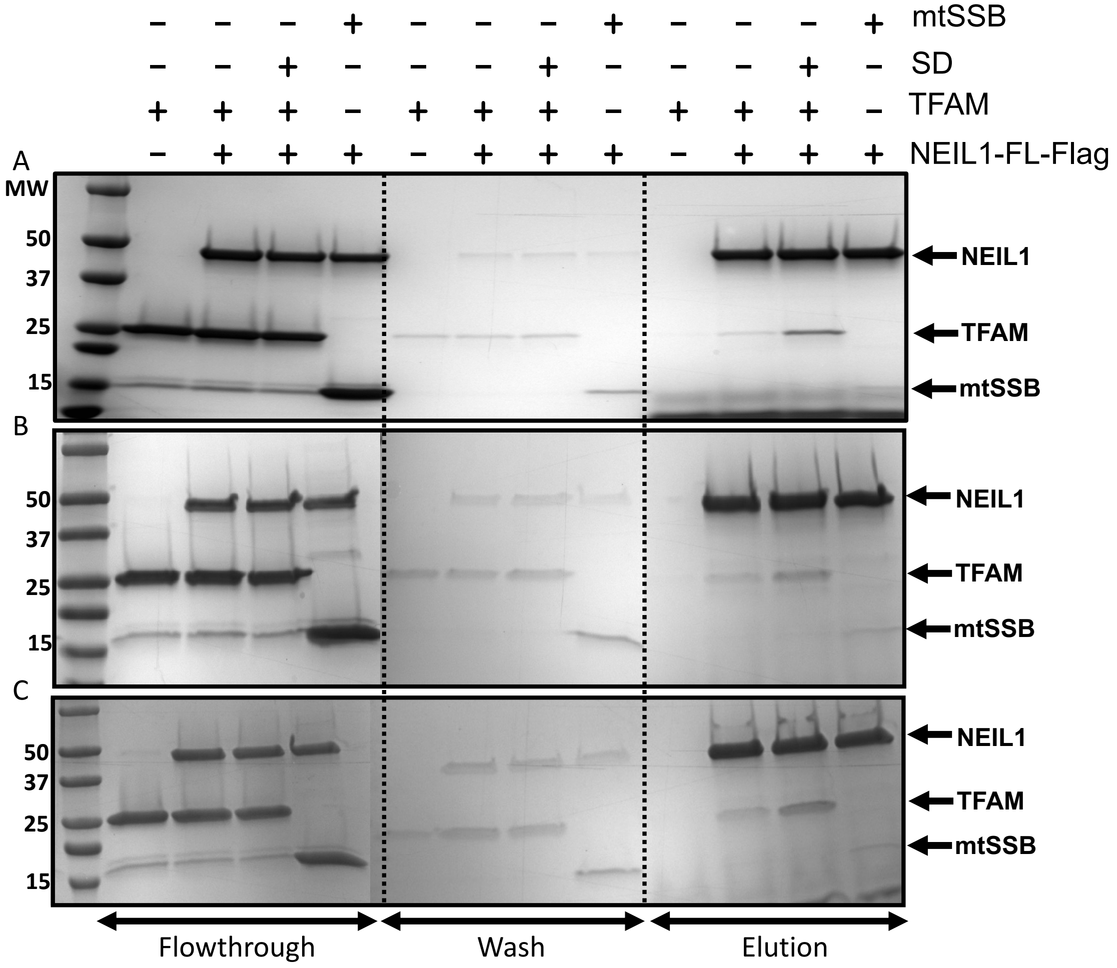

**Supplementary Figure 3:** Affinity pull-down experiments at 300 mM NaCl reveal a slight interaction between Flag-tagged NEIL1 and TFAM in the presence and absence of SD.

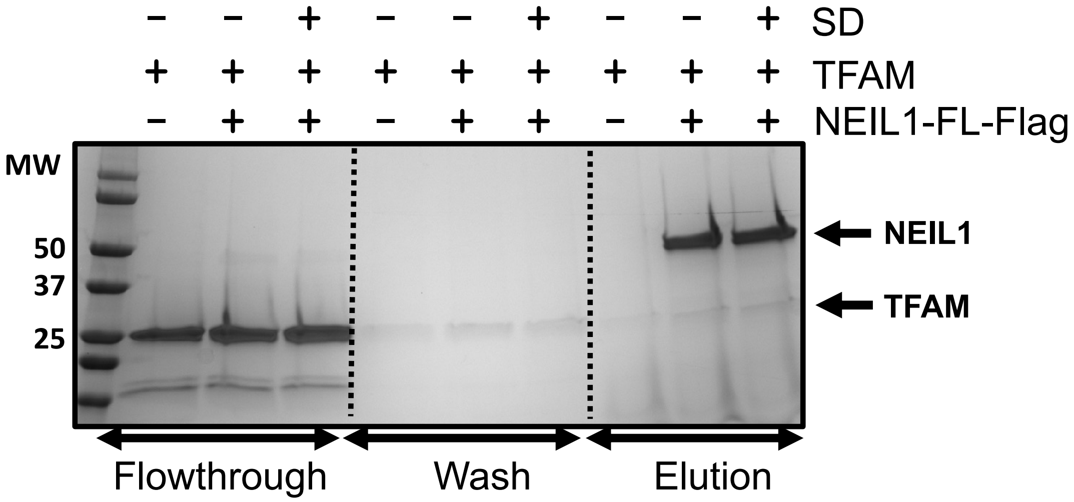

**Supplementary Figure 4:** Affinity flag pull-down experiments for flag tagged NEIL1 and TFAM at (A) 50 mM KCl, (B) 100 mM KCl, and (C) 150 mM KCl in the absence of nucleic acids.

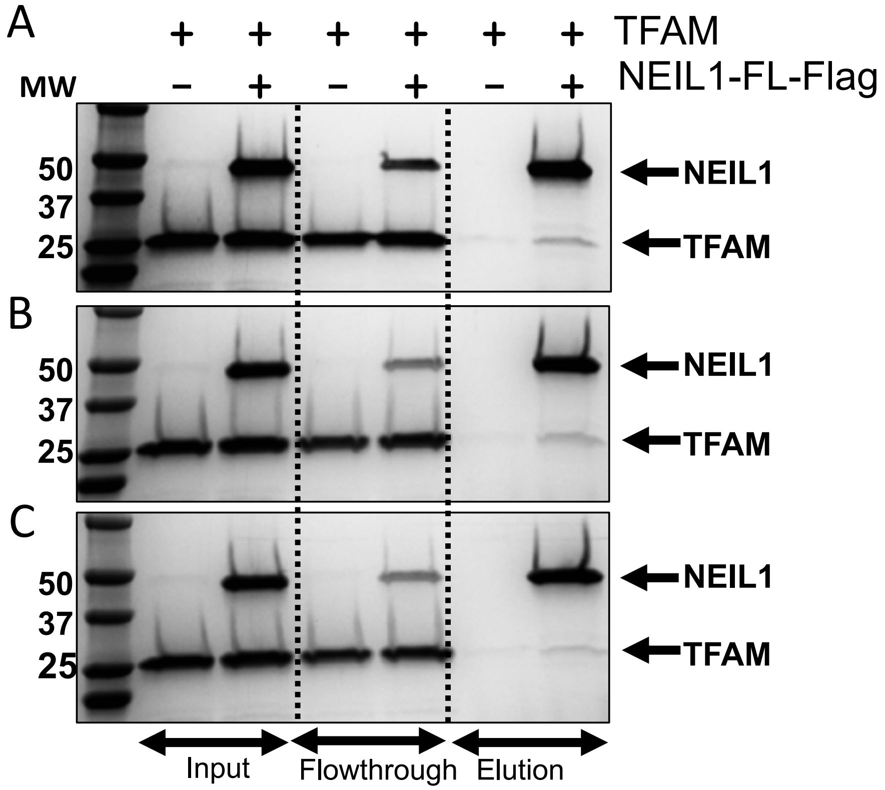

**Supplementary Figure 5:** Replicate gel analyses of the affinity flag pull-down experiments in the presence of RNA at (A) 100 mM NaCl and (B) 100 mM KCl.

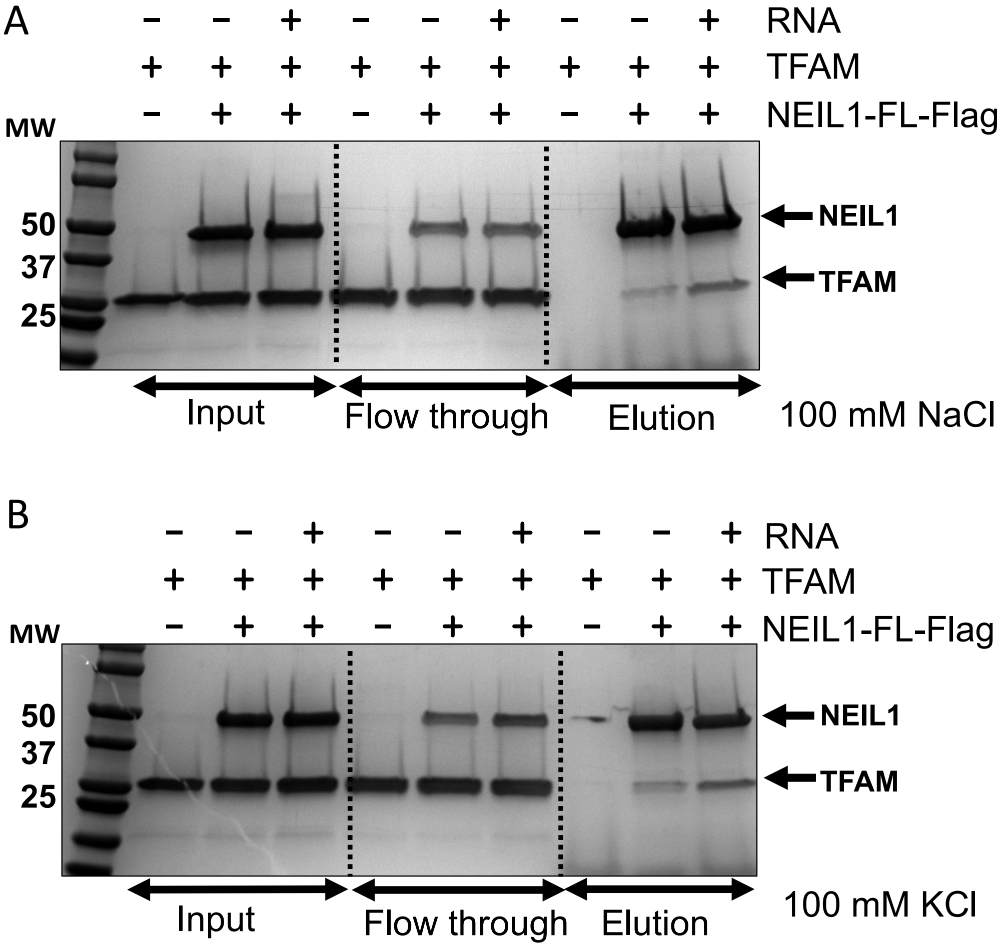

**Supplementary Figure 6:** Replicate gels of the far-western analysis displayed in Figure 3 of the main manuscript reveals that the interaction between NEIL1 and TFAM is mediated by residues present within the N and C-terminal domains of NEIL1.

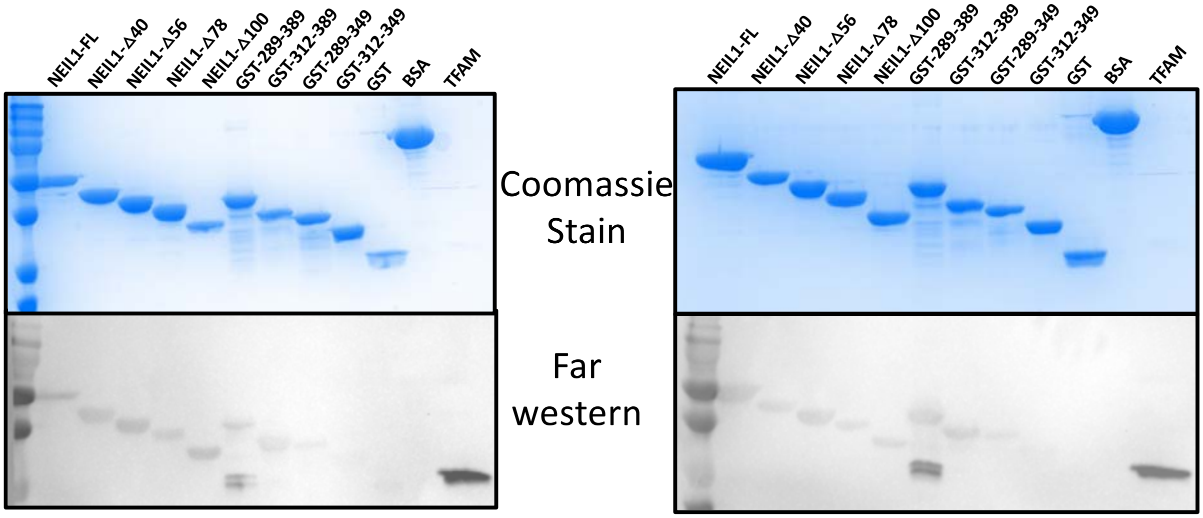

**Supplementary Figure 7**: Size-exclusion chromatography (SEC) to isolate complexes of NEIL1 and TFAM in the presence of DNA. **(A – F)** SEC elution profiles from a pre-calibrated Superdex 200 column for samples containing the NEIL1 and TFAM proteins alone, binary complexes of the proteins in the presence or absence of SD or NSD, or ternary complexes containing NEIL1-TFAM-SD or -NSD.

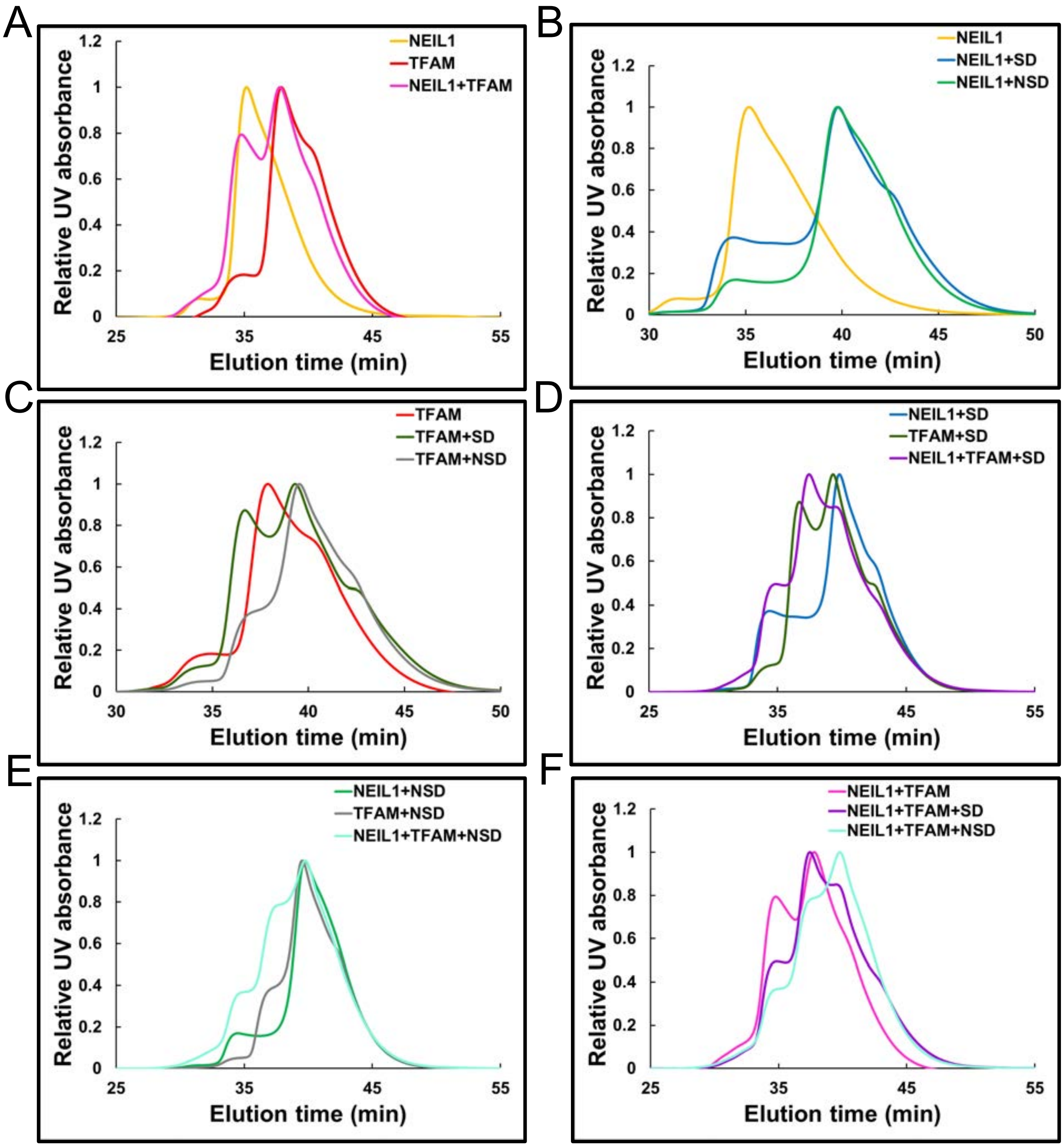

**Supplementary Figure 8:** SEC-SAXS analysis for NEIL1 **(A)**, TFAM **(B)**, NEIL1+TFAM **(C)**, NEIL1+SD **(D)**, NEIL1+NSD **(E)**, TFAM+SD **(F)**, TFAM+NSD **(G)**, TFAM+NEIL+SD **(H)**, TFAM+NEIL1+NSD **(I)**. Evolving factor analysis was performed during SAXS data processing to extract multiple components. For each protein/sample mixture, the SEC profile from the Superdex 200 column is displayed in the top left panel, the SAXS scattering curve is at the top right, pairwise distance distribution function is displayed on the bottom left, and the dimensionless (Rg) Kratky plot is on the bottom right. I- scattering intensity, q-momentum transfer, Rg - radius of gyration, and P(r) - pairwise distance distribution function.

Supplementary Figure 8A: NEIL1

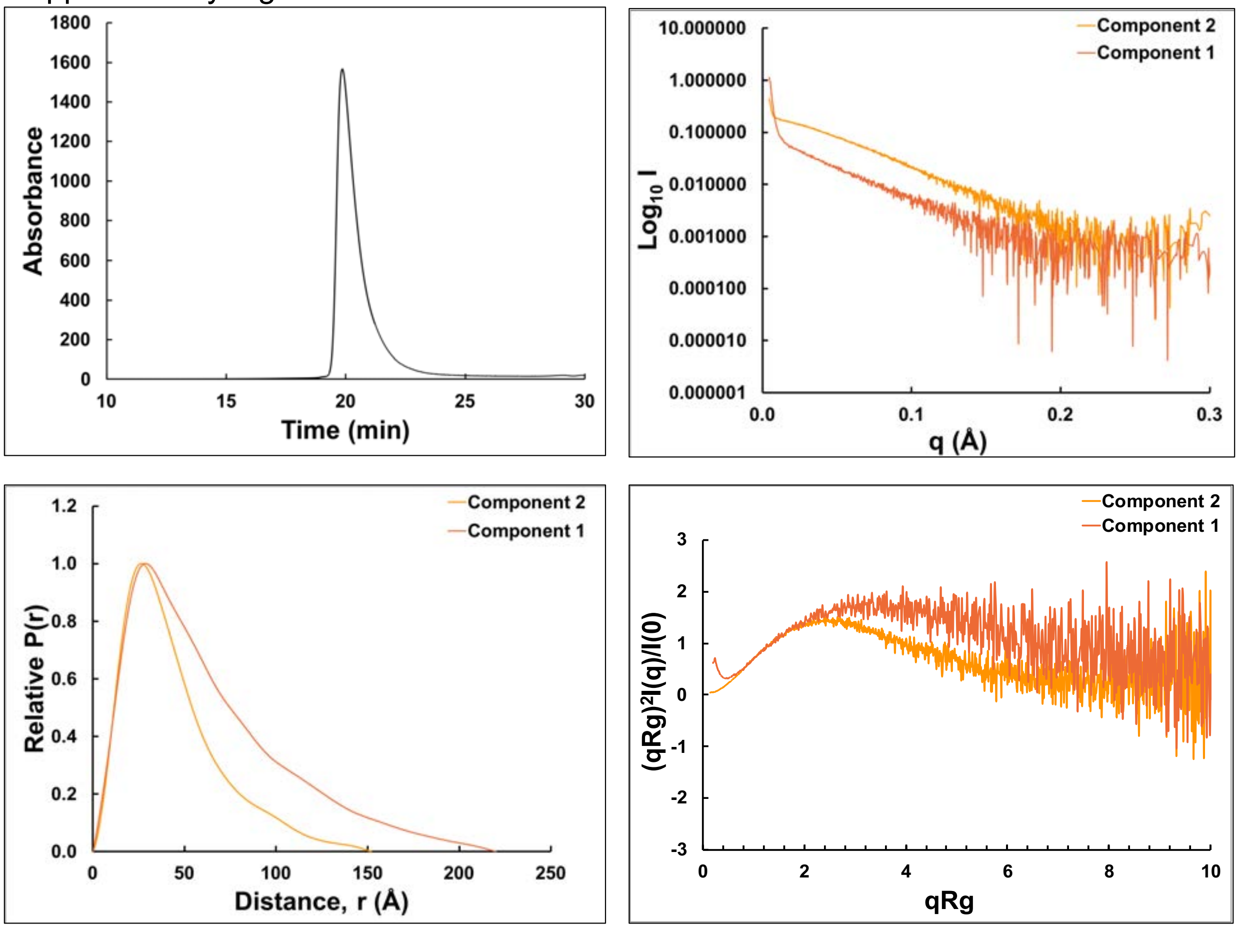

Supplementary Figure 8B: TFAM

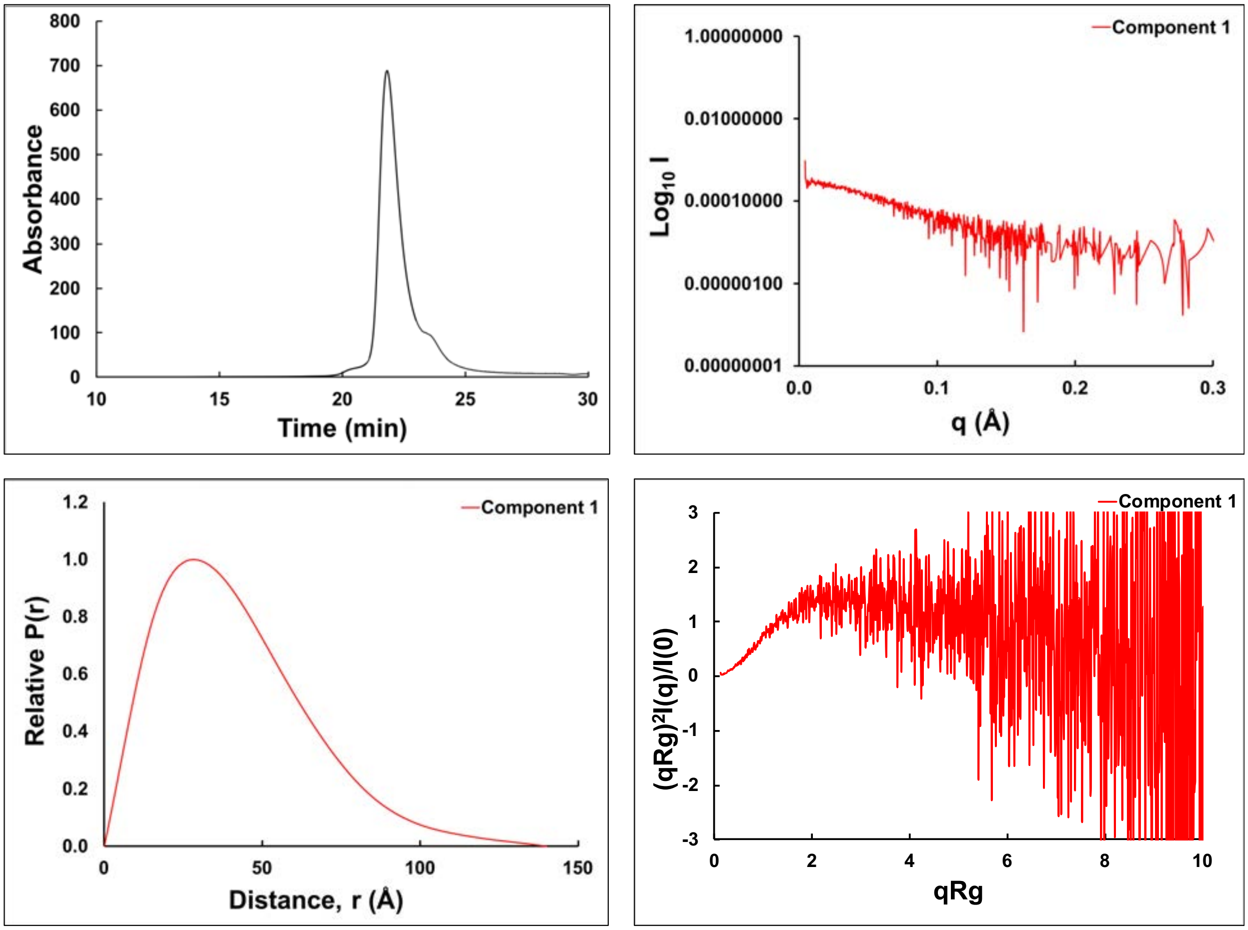

Supplementary Figure 8C: NEIL1 + TFAM

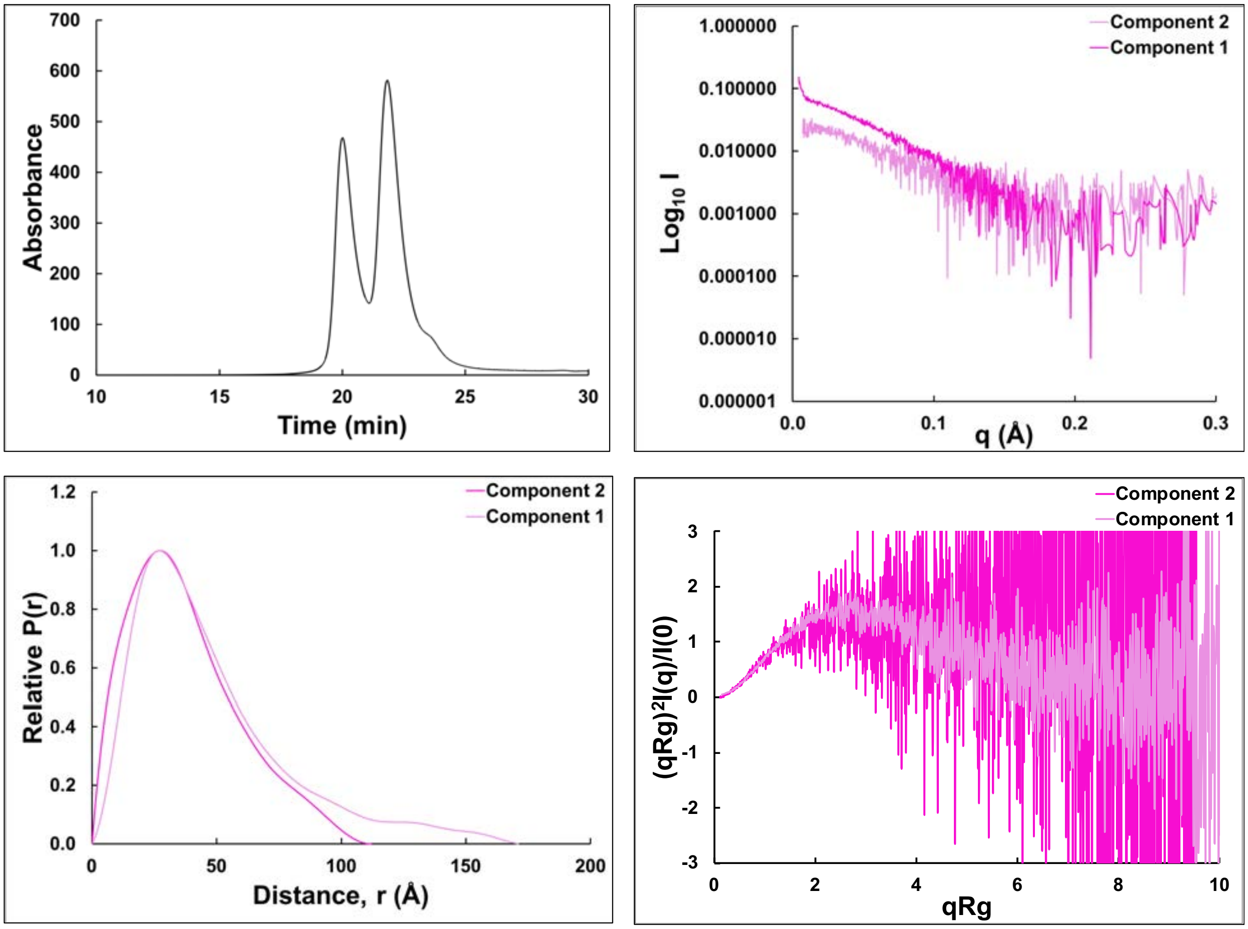

Supplementary Figure 8D: NEIL1 + SD

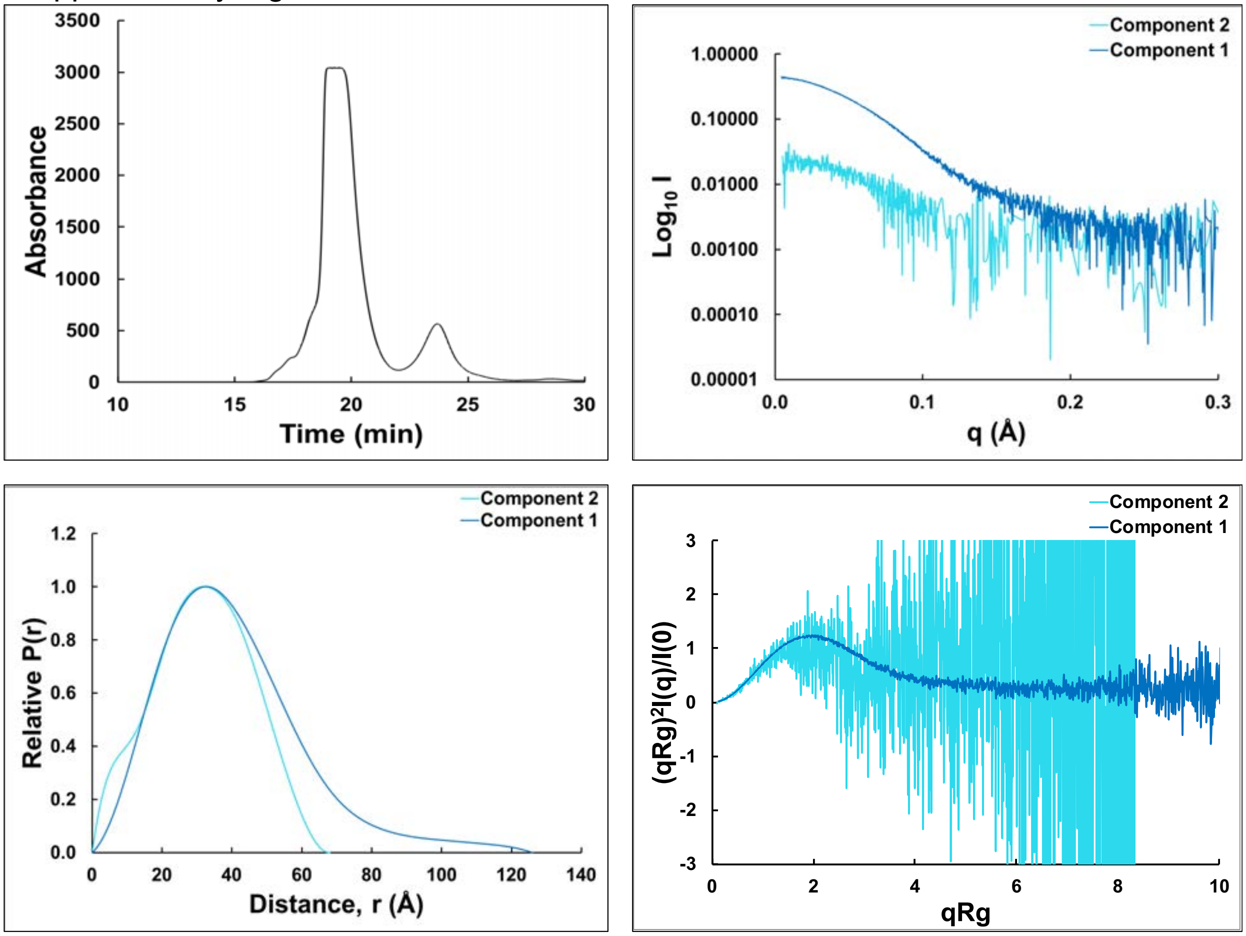

Supplementary Figure 8E: NEIL1 + NSD

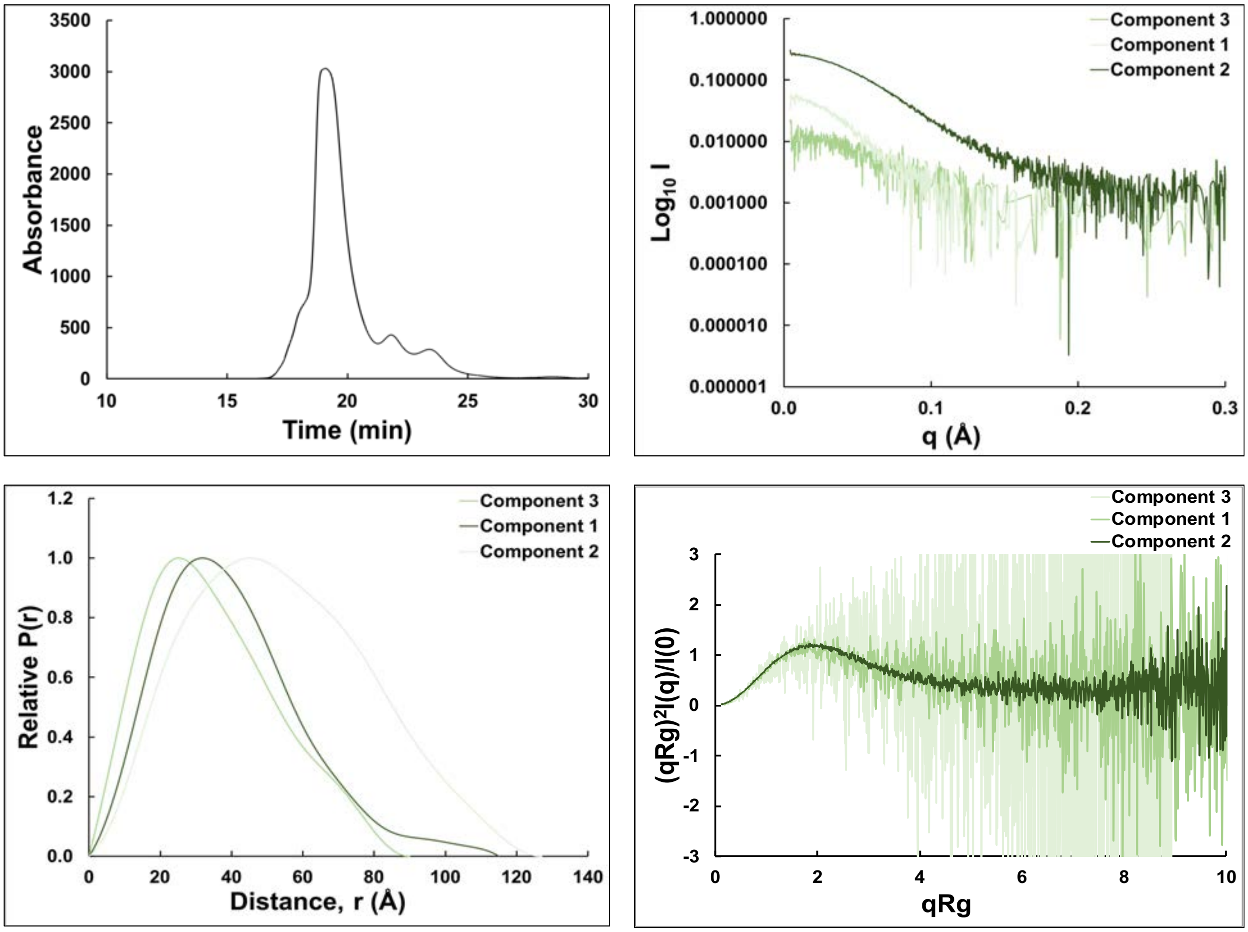

Supplementary Figure 8F: TFAM + SD

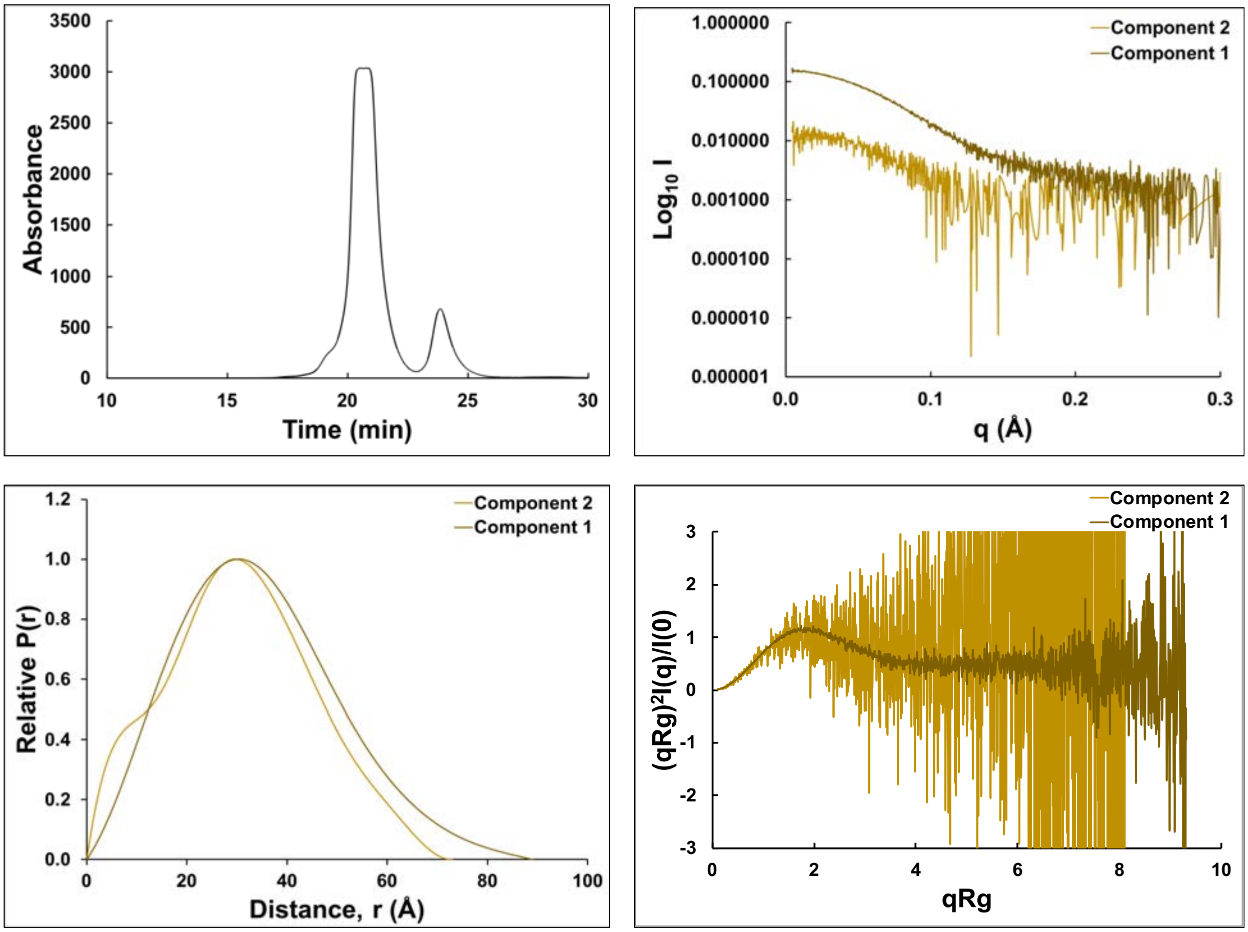

Supplementary Figure 8G: TFAM + NSD

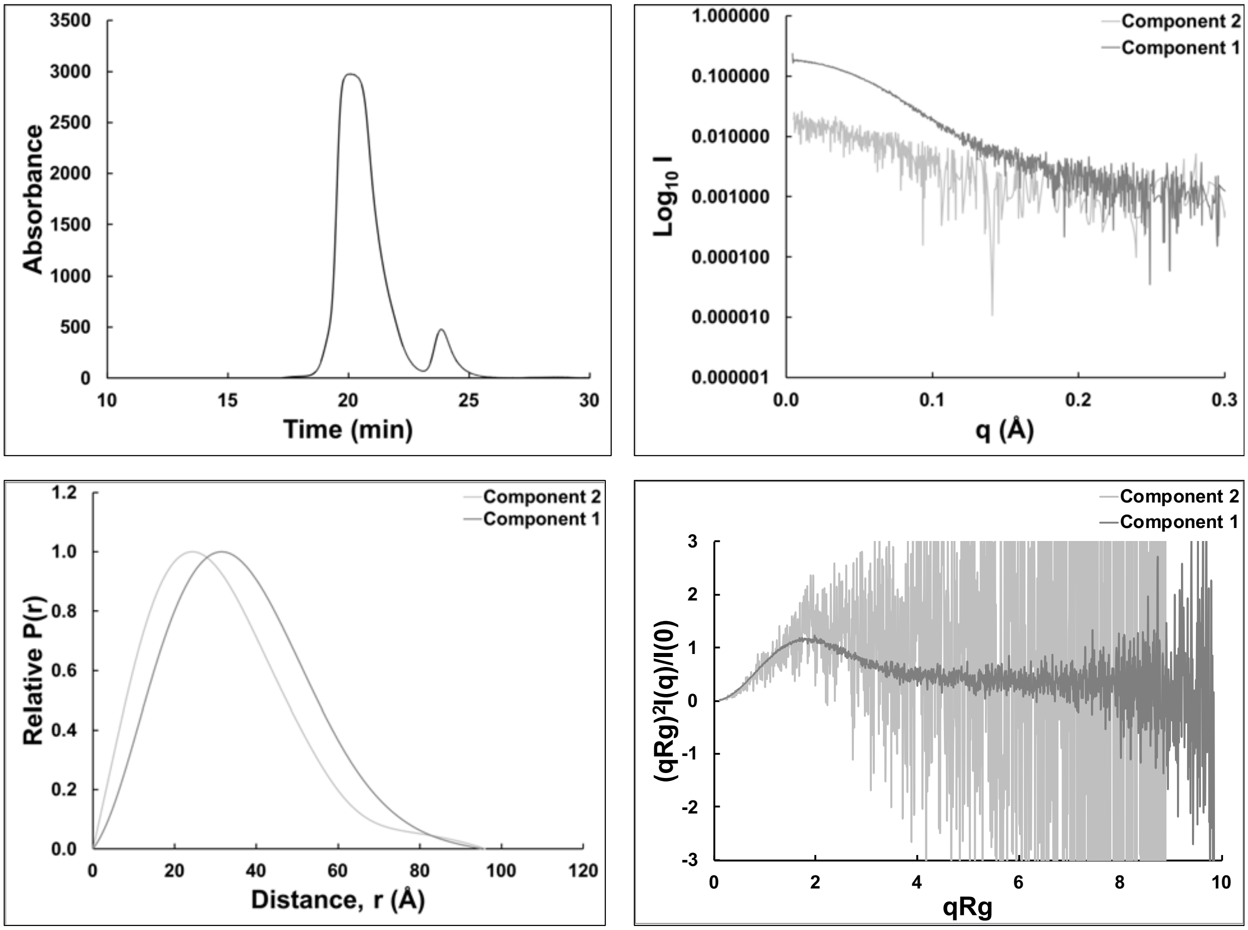

Supplementary Figure 8H: TFAM + NEIL1 + SD

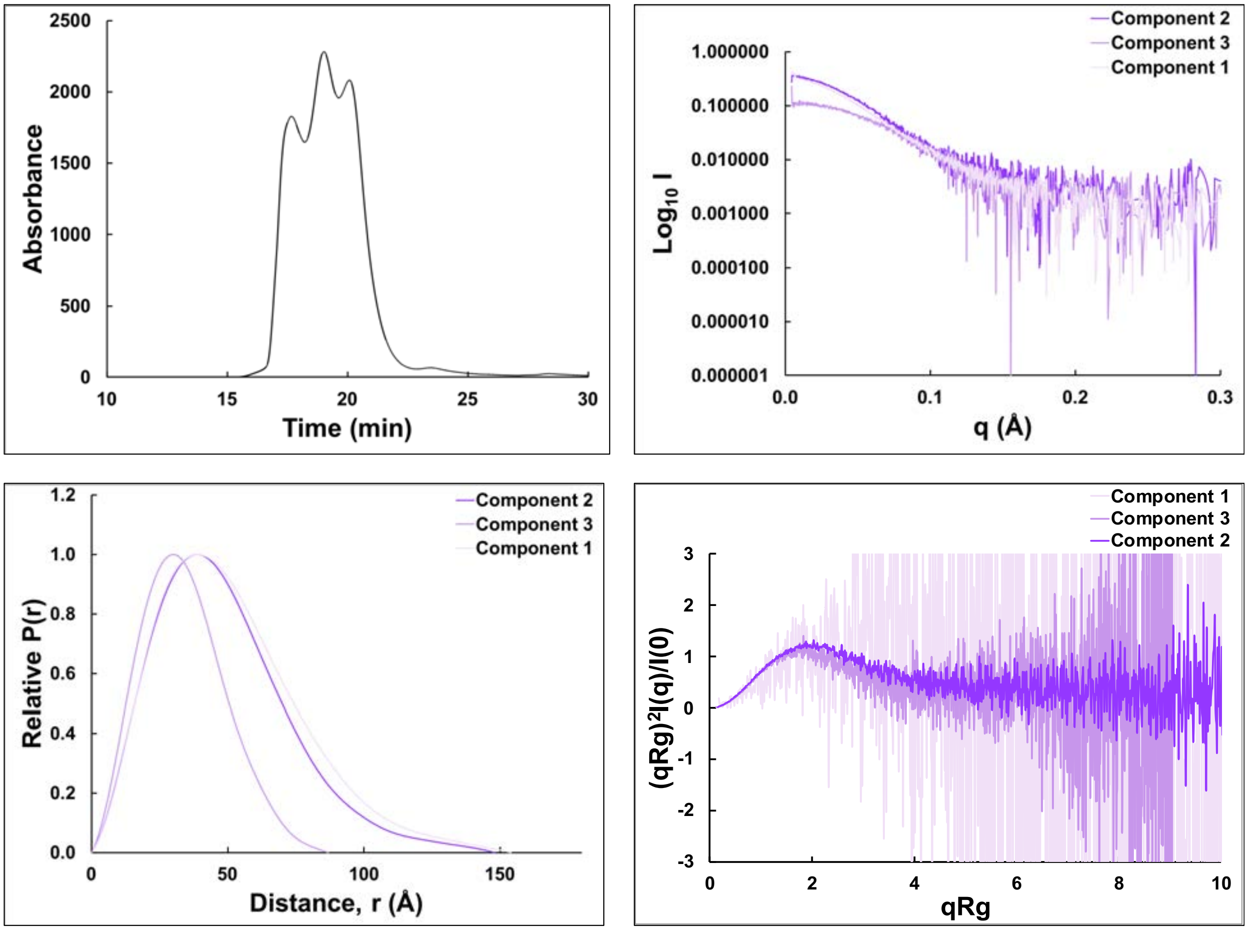

Supplementary Figure 8I: TFAM + NEIL1 +NSD

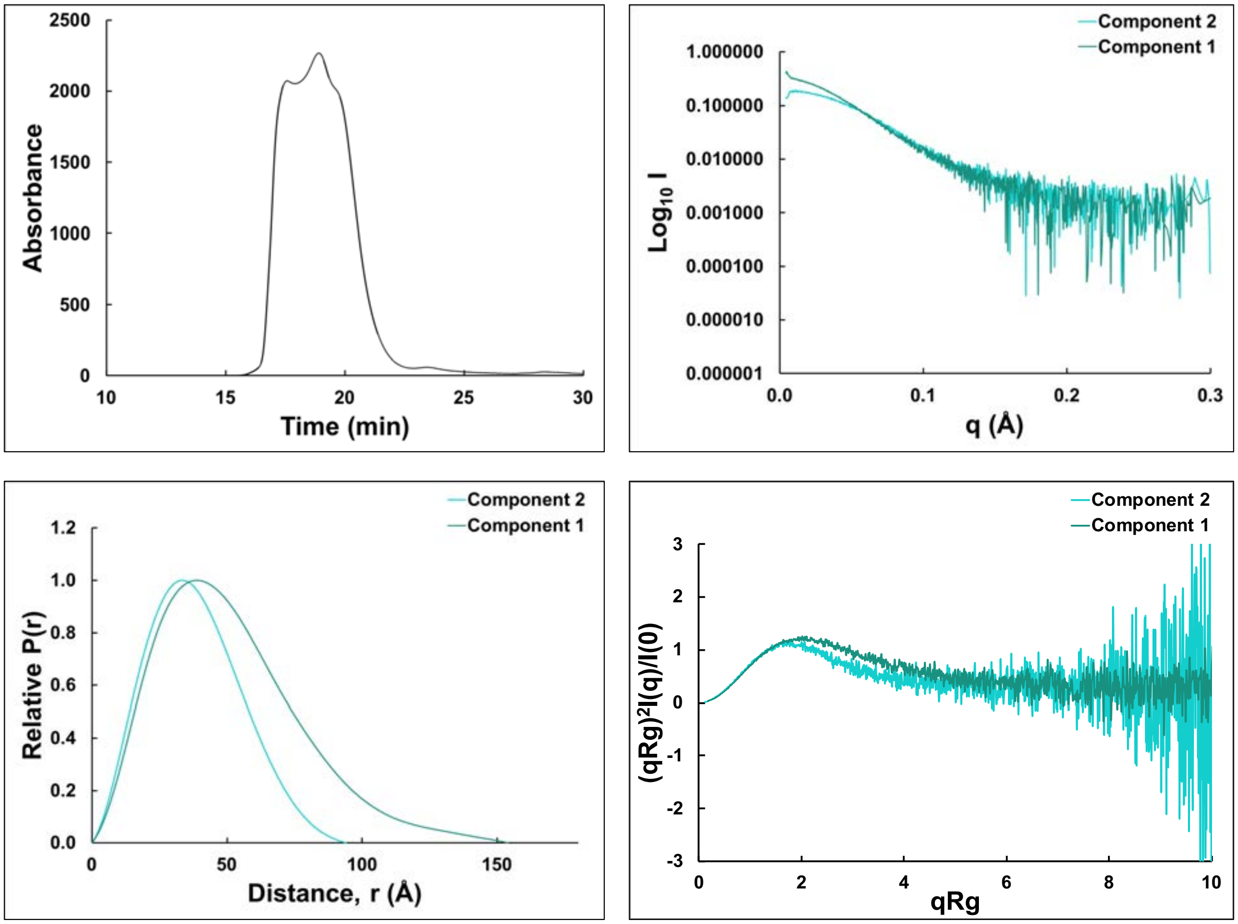

**Supplementary Figure 9**: Coverage maps for pepsin digested TFAM peptides in the TFAM, TFAM-DNA, and TFAM-NEIL1-DNA samples after HDX. Each colored bar represents a digested peptide monitored during the experiment. The colors indicate average deuterium uptake differences where red represents peptides with the highest uptake and blue corresponds to the lowest uptake. Peptide coverage of the protein sequence for each sample indicated 94.86% coverage for TFAM, 84.58% for TFAM-DNA, and 94.86% for the TFAM-NEIL1-DNA complex.

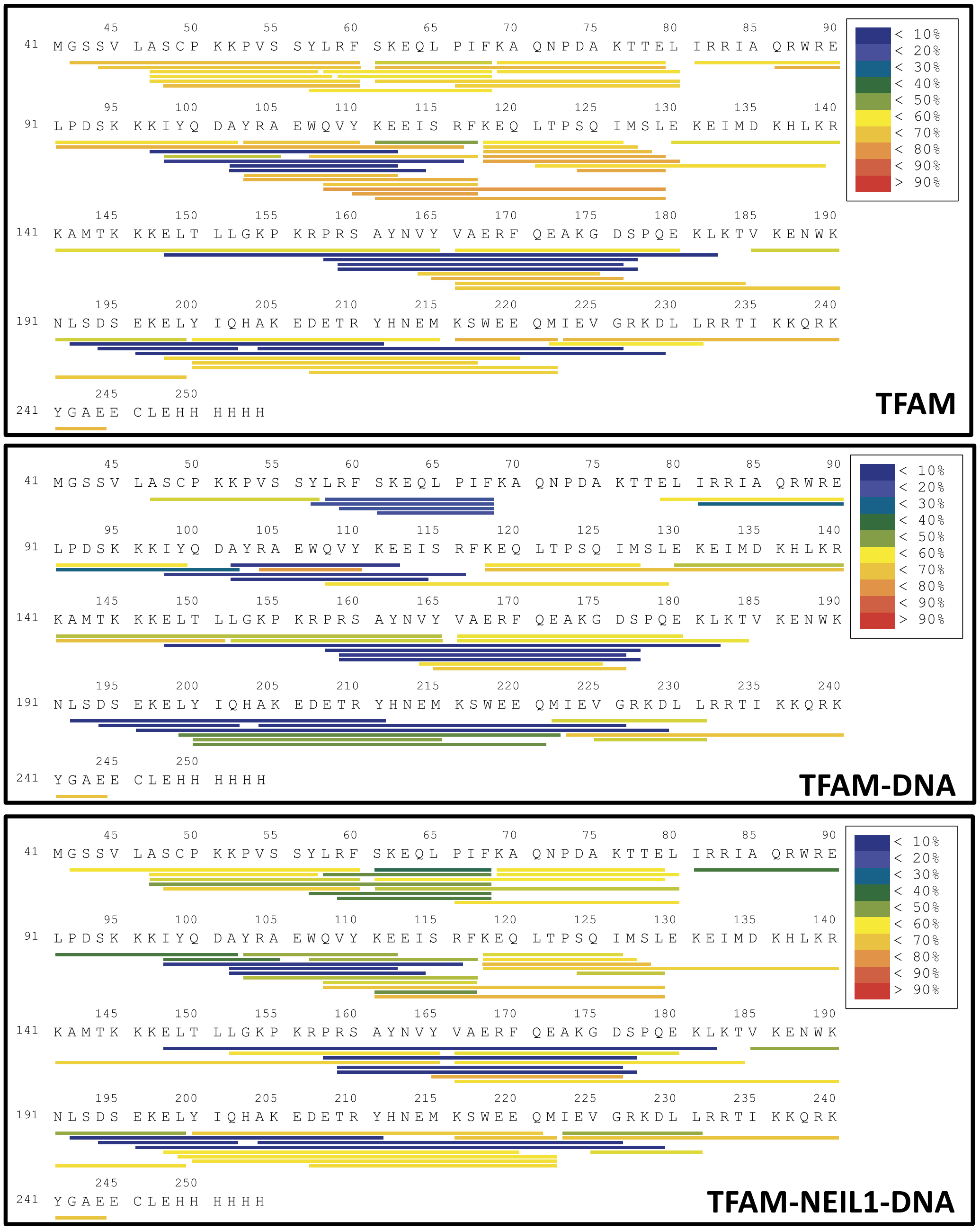

**Supplementary Figure 10**: Coverage maps for pepsin digested NEIL1 **(A)** and NEIL1-DNA **(B)** after HDX. Peptide coverage of the protein sequence for each sample indicated 98.74% coverage for both NEIL1 and NEIL1-DNA.

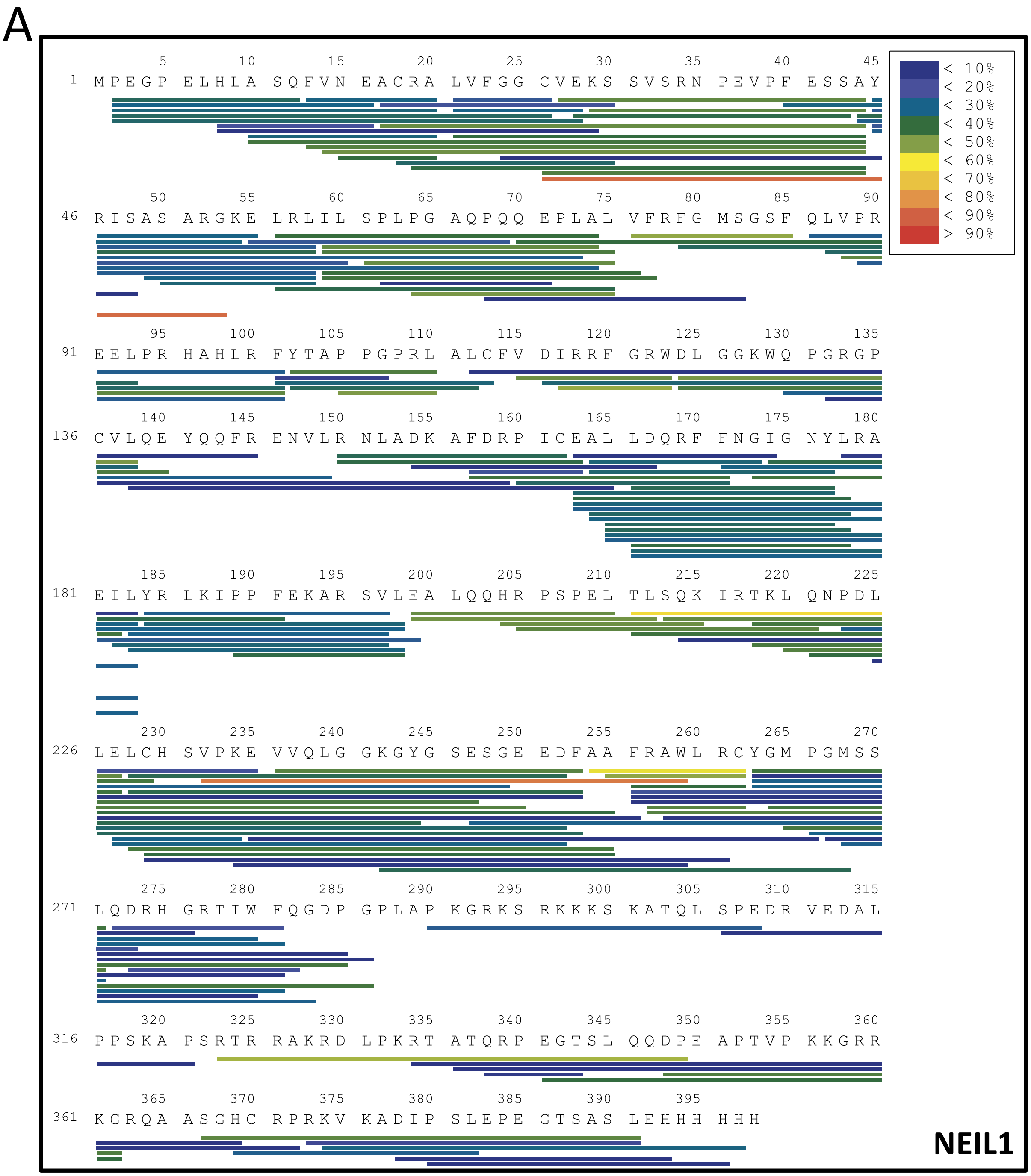

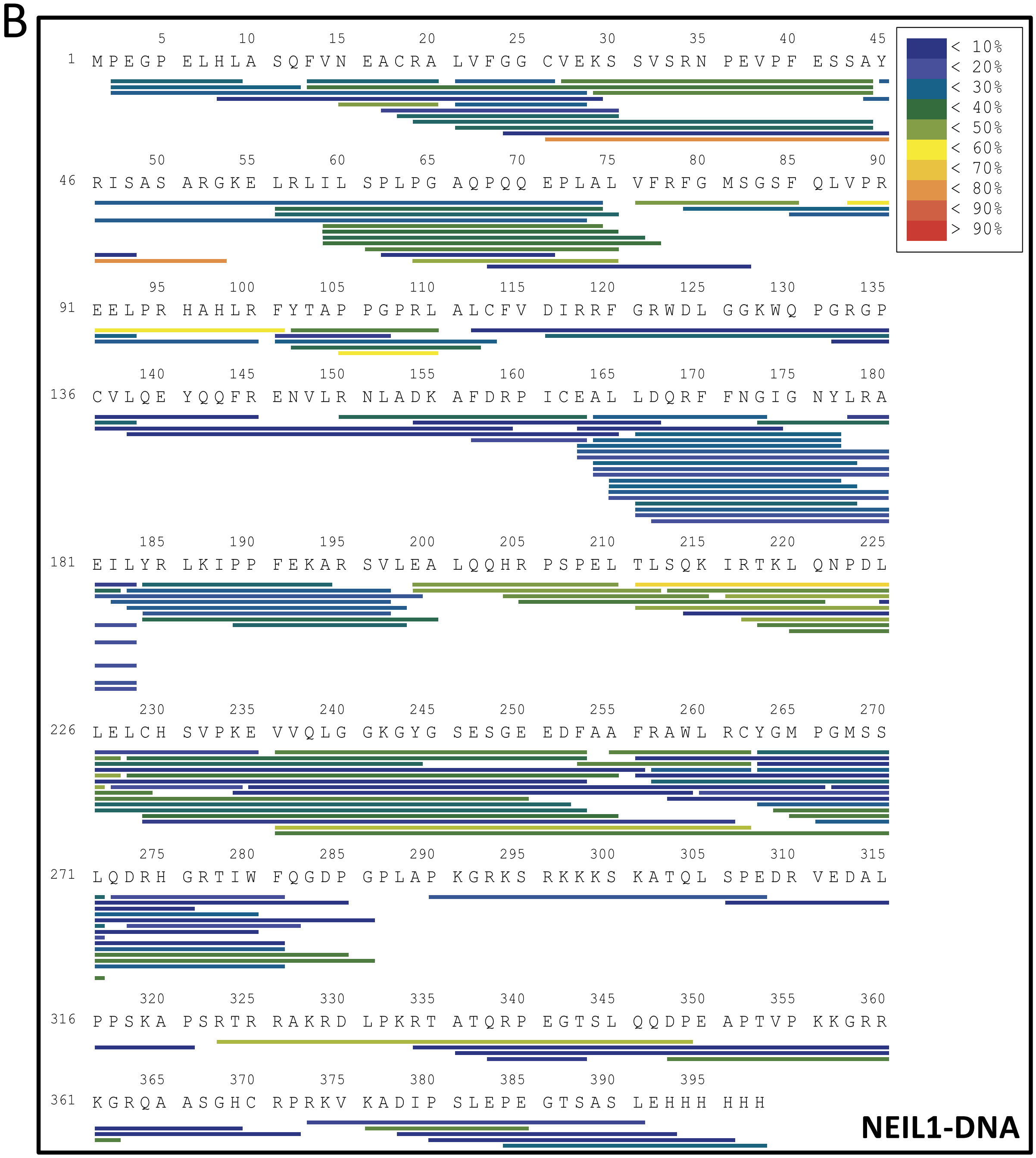
B
1 MPEGP ELHLA SQFVN EACRA LVFGG CVEKS SVSRN PEVPF ESSAY
46 RISAS ARGKE LRLIL SPLPG AQPQQ EPLAL VFRFG MSGSF QLVPR
91 EELPR HAHLR FYTAP PGPRL ALCFV DIRRF GRWDL GGKWQ PGRGP
136 CVLQE YQQFR ENVLR NLADK AFDRP ICEAL LDQRF FNGIG NYLRA
181 EILYR LKIPP FEKAR SVLEA LQQHR PSPEL TLSQK IRTKL QNPDL
226 LELCH SVPKE VVQLG GKGYG SESGE EDFAA FRAWL RCYGM PGMSS
271 LQDRH GRTIW FQGDP GPLAP KGRKS RKKKS KATQL SPEDR VEDAL
316 PPSKA PSRTR RAKRD LPKRT ATQRP EGTSL QQDPE APTVP KKGRR
361 KGRQA ASGHC RPRKV KADIP SLEPE GTSAS LEHHH HHH
< 10%
< 20%
< 30%
< 40%
< 50%
< 60%
< 70%
< 80%
< 90%
> 90%
NEIL1-DNA

**Supplementary Figure 11:** Isotopic mass distribution spectra for the TFAM peptide comprising residues 57-68 at the 30s timepoint. The grey and blue colors represent experimental and theoretical clusters. The orange and green colors represent two differentially exchanged peptides when the data is fitted using bimodal distribution analysis.

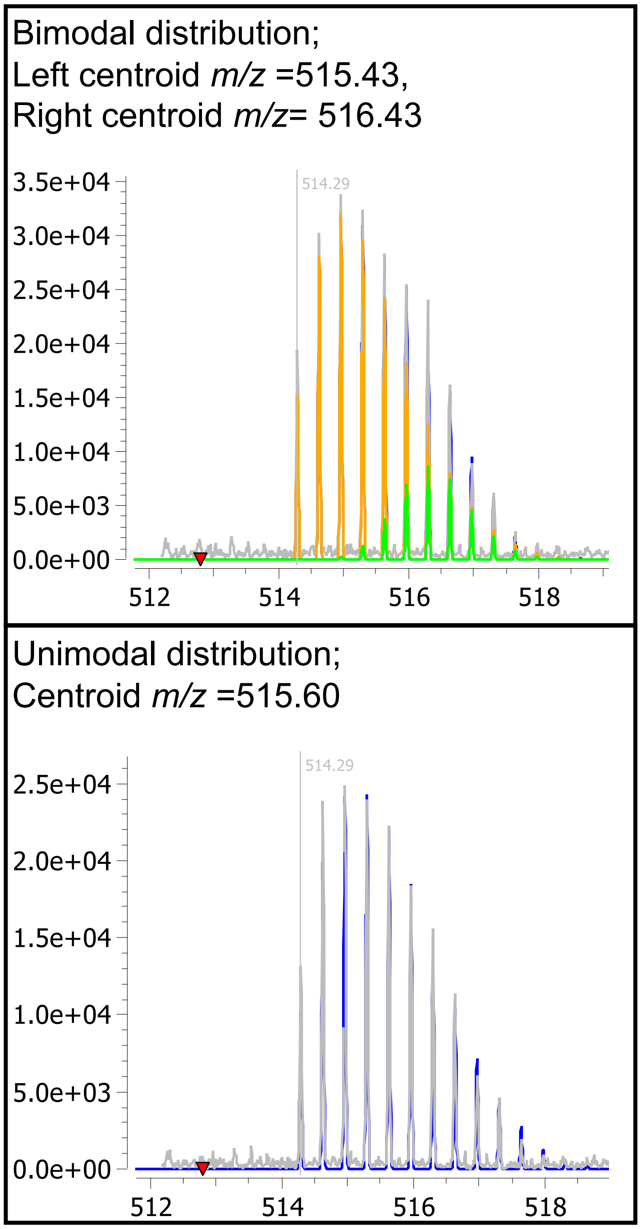

**Supplementary Figure 12:** NEIL1 expression and cell survival. **(A)** Western blot analysis for NEIL1 expression in WT and KO Hap1 cells indicates no NEIL1 expression in the KO cell line. PCNA expression was used as the loading control. **(B)** Dose-response curves to assess Hap1 cell survival plotted as the relative fluorescent units (RFU) generated using a resazurin-based fluorescent assay versus the final concentration of MMS. Lines represent non-linear regression analysis ([Inhibitor] vs. response) performed using GraphPad Prism 8.

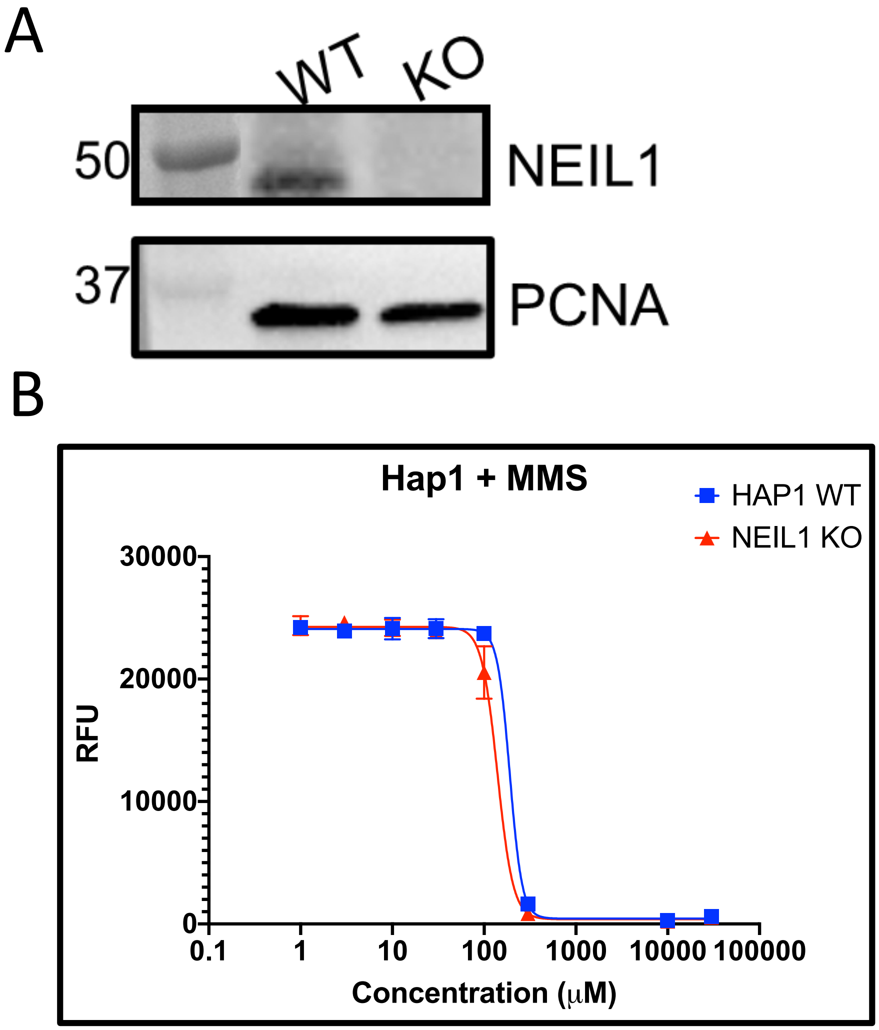

Supplement: Supplementary file 1 [file DataSheet1.PDF]
